# Tubular epithelial cells in renal clear cell carcinoma express high RIPK1/3 and show increased susceptibility to TNF receptor 1-induced necroptosis

**DOI:** 10.1038/cddis.2016.184

**Published:** 2016-06-30

**Authors:** R S Al-Lamki, W Lu, P Manalo, J Wang, A Y Warren, A M Tolkovsky, J S Pober, J R Bradley

**Affiliations:** 1 Department of Medicine, University of Cambridge, Addenbrooke's Hospital, Cambridge CB2 0QQ, UK; 2 Department of Pathology, University of Cambridge, Addenbrooke's Hospital, Cambridge CB2 0QQ, UK; 3 Department of Clinical Neurosciences, University of Cambridge, Addenbrooke's Hospital, Cambridge CB2 0QQ, UK; 4 Department of Immunobiology, Yale University School of Medicine, New Haven, CT 06510, USA

## Abstract

We previously reported that renal clear cell carcinoma cells (RCC) express both tumor necrosis factor receptor (TNFR)-1 and -2, but that, in organ culture, a TNF mutein that only engages TNFR1, but not TNFR2, causes extensive cell death. Some RCC died by apoptosis based on detection of cleaved caspase 3 in a minority TUNEL-positive cells but the mechanism of death in the remaining cells was unexplained. Here, we underpin the mechanism of TNFR1-induced cell death in the majority of TUNEL-positive RCC cells, and show that they die by necroptosis. Malignant cells in high-grade tumors displayed threefold to four fold higher expression of both receptor-interacting protein kinase (RIPK)1 and RIPK3 compared with non-tumor kidney tubular epithelium and low-grade tumors, but expression of both enzymes was induced in lower grade tumors in organ culture in response to TNFR1 stimulation. Furthermore, TNFR1 activation induced significant MLKL^Ser358^ and Drp1^Ser616^ phosphorylation, physical interactions in RCC between RIPK1-RIPK3 and RIPK3-phospho-MLKL^Ser358^, and coincidence of phospho-MLKL^ser358^ and phospho-Drp1^Ser616^ at mitochondria in TUNEL-positive RCC. A caspase inhibitor only partially reduced the extent of cell death following TNFR1 engagement in RCC cells, whereas three inhibitors, each targeting a different step in the necroptotic pathway, were much more protective. Combined inhibition of caspases and necroptosis provided additive protection, implying that different subsets of cells respond differently to TNF-*α*, the majority dying by necroptosis. We conclude that most high-grade RCC cells express increased amounts of RIPK1 and RIPK3 and are poised to undergo necroptosis in response to TNFR1 signaling.

Renal clear cell carcinoma (RCC) is resistant to chemotherapy and 5-year survival rates of metastatic disease are only 5–15%.^1^ Many anticancer agents act via induction of apoptosis, and apoptotic deficiency may be a cause of chemoresistance. However, recent studies have identified a caspase-independent form of programmed cell death, termed necroptosis,^2^ that may provide an alternative pathway for tumor killing.^3, 4^ Tumor necrosis factor-*α* (TNF), acting through TNF receptor 1 (TNFR1), induces apoptosis of many different types of malignant cells through caspase 8 activation, which is mediated through the assembly of a signaling complex involving TNFR-associated protein with a death domain (TRADD), receptor-interacting protein kinase 1 (RIPK1), TNFR-associated factor 2 (TRAF2) and Fas-associated protein with a death domain (FADD).^5^ Alternatively, TNF can induce necroptosis, also via TNFR1, through a pathway involving TRADD, RIPK1 and RIPK3.^2, 6, 7, 8, 9, 10^ Signaling through TNFR2, which is also expressed on RCC cells, does not generally induce cell death, but can potentiate TNFR1-mediated programmed necrosis via TNFR1.^11^ Activation of caspase 8 generally inhibits death by necroptosis so that necroptosis is more evident when caspase activation is inhibited or in cells lacking FADD or caspase 8.^12, 13^ In necroptosis, RIPK1 is recruited to TRADD via a death domain (DD) located in its C terminus^14, 15^ and RIPK1 then recruits RIPK3, forming a necroptosis-inducing 'ripoptosome complex'^16^ that phosphorylates pseudokinase mixed lineage kinase domain-like (MLKL) at residues threonine 357 and serine 358 in humans^15, 17, 18, 19^ and serine 345 in mice.^20^ The events that follow may be cell type-specific, but in some cells, this promotes MLKL association with the mitochondrial phosphatase PGAM5 and dyamin-related protein 1 (Drp1), a cytosolic dynamin GTPase. Dephosphorylation of Drp1 at serine 637 by PGAM5 results in Drp1 activation,^15, 21, 22^ which leads to mitochondrial fragmentation and necroptosis.^23^ Loss of Drp1 function has been shown to slow down necrosis in *Caenorhabditis elegans*^24^ and inhibition of its activity delays cell death.^25^ Phosphorylation of serine 616 promotes Drp1 activity and tumor growth.^26^ This new understanding of the signal transduction pathway leading to necroptosis offers the opportunity to target this process therapeutically. We have used organ culture to study the responses of RCC cells to TNF signaling.^5^ The advantage of using organ culture is that it preserves the relationship between the various cell types within the cancer milieu and also preserves adjacent normal tissue, which can be used as an endogenous control. We previously reported that ligation of TNFR1 promotes cell death in malignant tubular epithelial cells (mTECs) in RCC, which we had attributed to apoptosis based on detection of cleaved caspase 3 in some of the TUNEL-positive cells.^27^ These initial studies antedated the elucidation of the necroptotic process. In the present study, we show that both apoptosis and necroptosis occur within the same tumor and that necroptosis may be the dominant pathway of cell death in TNF-treated RCC.

## Results

### RIPK1 and RIPK3 are highly expressed in RCC and are upregulated by TNF

**Figure 1:**
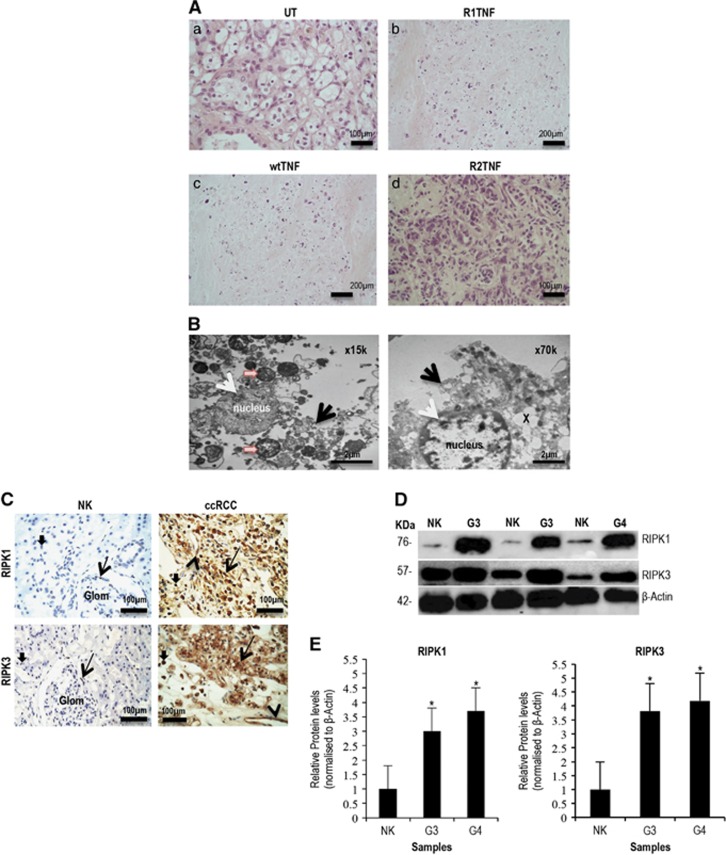
(**A**) Histology of untreated cultures (UT) show (a) low-grade RCC with small round tumor cells and a clear cytoplasm surrounded by a distinct cell membrane and round uniform nuclei with inconspicuous or absent nucleoli. (b) R1TNF- and (c) wtTNF-treated cultures show elevated level of death in mTECs compared with (d) R2TNF-treated cultures. (**B**) Morphological features of necrosis are evident in R1TNF-treated cultures such as large/small cytoplasmic vacuoles (x), condensation of the chromatin into small, irregular, circumscribed patches (white arrow), increasing translucent cytoplasm, swollen organelles (orange arrow) and disruption of the plasma membrane (black arrows). (**C**) Expression of RIPK1 and RIPK3 in tissue biopsies comprising RCC and NK; NK show a rare signal for RIPK1 and RIPK3 in MNCs within glomeruli (arrows) and with interstitium (small arrows). In contrast, RCC grade 3 shows marked signal in mTECs (arrows), MNCs (small arrows) and in some VECs (arrowheads). (**D** and **E**) Representative immunoblot of samples from nine patients with similar results and bar graph of relative RIPK1 and RIPK3 protein levels in grades 3/4 RCC (G3/G4) and non-tumor kidney (NK). Bars=mean+S.E.M.; *P<0.05 *versus* NK; paired Student's *t*-test. Glom-glomeruli; MNCs-mononuclear cells; TECs-tubular epithelial cells

In an earlier report,^27^ we showed that ~10% of RCC cells in organ culture die by apoptosis in response to TNF, via the TNFR1 receptor. However, we noted that more cells died than those accounted for by apoptosis alone. One possible explanation is that some of the cells were dying by necroptosis. To study whether RCC cells die by necroptosis, we analyzed RCC cells more thoroughly for signs of necrosis. There was about 70–80% cell death in RCC organ cultures treated with wtTNF or R1TNF compared with the control group (UT) or R2TNF-treated cultures (Figure 1A). A closer examination by electron microscopy identified features of necrosis in mTECs such as numerous large and small cytoplasmic vacuoles, condensation of the chromatin into small, irregular, circumscribed patches, increased translucent cytoplasm, swelling of organelles and disruption of the plasma membrane^28^ (Figure 1B). Many of the necrotic cells contained TUNEL^+^ nuclei, indicating DNA fragmentation, a feature widely associated with apoptosis but also well documented to occur in primary necrosis.^29, 30^ Although TECs were the majority of cells displaying necrosis, some vascular endothelial cells and infiltrating mononuclear cells (MNCs) were also TUNEL^+^ (Supplementary Figure 1A, quantified in Supplementary Figure 1B).

TNF is known to induce necroptosis via RIPK1/3.^6, 15, 19, 31-33^ To determine whether the necrosis observed may be due to necroptosis, we determined whether RIPK1 and RIPK3 are expressed in clinical samples of RCC prior to organ culture, using adjacent histologically normal non-neoplastic kidney (NK) as controls. Sections of NK demonstrated normal intact tubules and remarkable glomeruli. In contrast, sections of RCC grades 1–4 demonstrated neoplastic morphology with increasing necrotic features, RCC grade 4 displaying extensive areas of necrosis, with nuclear material embedded within necrotic zones (Supplementary Figure 2). Remarkably, a strong homogenous expression of RIPK1 and RIPK3 in >60% of cells was observed in high-grade tumors (3/4), mainly confined to mTECs, and a few infiltrating MNCs and vascular endothelial cells (Figure 1C). In contrast, both proteins were detected only in MNCs within glomerular and interstitium in NK. Staining for RIPK1 was mainly cytoplasmic, whereas RIPK3 was cytoplasmic and nuclear. Immunoblotting of tissue lysates was concordant with IHC findings, showing a threefold to fourfold increase in both RIPK1 and RIPK3 expression in grade 3–4 RCC (Figure 1D, quantified in Figure 1E). No obvious difference in staining pattern was observed with all the RIPK1 and RIPK3 antibodies.

**Figure 2:**
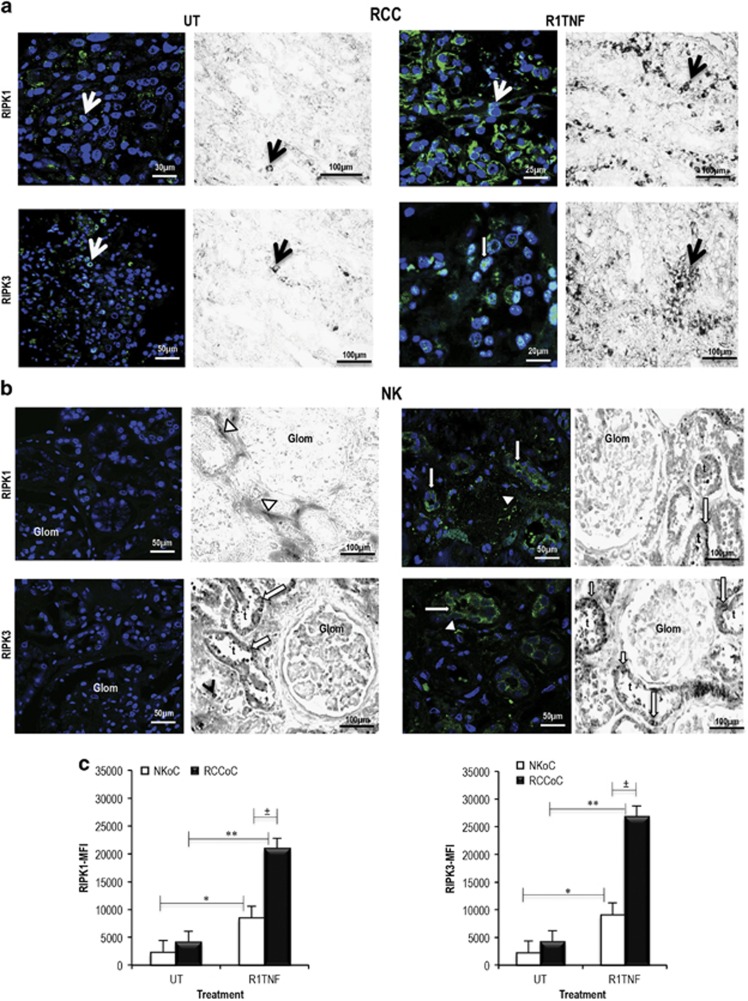
Representative confocal images and light micrographs of the effect of R1TNF on protein and mRNA expression for RIPK1 and RIPK3 in organ cultures of RCC grade 1 and adjacent non-tumor kidney (NK). (**a** and **b**) Untreated (UT) cultures from RCC show a rare signal for RIPK1 and RIPK3 protein and mRNA. In contrast, R1TNF-treated cultures show a marked signal of both proteins mainly confined to mTECs (arrows), with RIPK3 also present in nuclei (white shaded arrow). Similarly, UT cultures of NK show a rare signal for both proteins; increased expression of both protein and mRNA is detected in R1TNF-treated cultures mainly confined to normal TECs (white arrows), peritubular capillaries (white arrowheads) and in infiltrating mononuclear cells (black arrowhead) but not in glomeruli (Glom). (**c**) Representative mean fluorescence intensity (MFI) for RIPK1 and RIPK3 expression in RCC and NK organ cultures. ^**^P<0.01 *versus* UT, *P<0.05 *versus* UT, ^±^*P*<0.05 *versus* R1TNF; Bars=mean±S.E.M.; *n*=3 independent experiments from six separate organ culture experiments with similar results. Nuclei stained with Hoechst 33342. Confocal images: × 40 and × 63 original magnifications; photomicrographs: × 400 magnification

To determine whether TNF regulates expression of RIPK1 and RIPK3, and which receptor is involved, we treated organ cultures of NK and low-grade tumors (1/2), as these samples showed low levels of RIPK1 and RIPK3, with wtTNF, R1TNF or R2TNF. UT controls showed a rare signal for RIPK1 and RIPK3 in <2% mTECs and infiltrating MNCs but a strong expression of RIPK1 and RIPK3 in >60% of mTECs after treatment with R1TNF (Figure 2a and Supplementary Figure 3). RIPK3 showed a cytoplasmic and nuclear pattern of staining, whereas RIPK1 was mainly cytoplasmic similar to the endogenous pattern found in 3/4 grades RCC. Interestingly, R1TNF-induced marked upregulation of RIPK1 (~5-fold) and RIPK3 (~6-fold) in RCC compared with NK organ cultures (~3-fold for RIPK1 and ~3-fold for RIPK3) (Figures 2b and c and Supplementary Figures 4A and 4B). RIPK1 and RIPK3 mRNA were also induced by R1TNF mainly in mTECs (Figure 2a). For both assays and study groups, wtTNF treatment showed similar intensity and pattern of expression as R1TNF while R2TNF induced a weak to moderate signal (Supplementary Figure 3). No signal was detected in parallel sections when the primary antibody was pre-adsorbed with a blocking peptide prior to immunostaining or after gene knockdown experiments using human HEK293 cells, as described in the Methods section (Supplementary Figures 6A and 6B). In addition, no signal was detected on sections hybridized with corresponding sense probes (data not shown). Taken together, these data suggest that TNF upregulates RIPK1 and RIPK3 expression in mTECs in RCC predominantly via TNFR1, and that this response is more pronounced in RCC compared with adjacent NK. For assessment of whether increased RIPK1 and RIPK3 expression was dependent on NF*κ*B activation, organ cultures were pretreated with pyrrolidine dithiocarbamate (PDTC),^34^ an inhibitor of NF*κ*B activation, before TNF treatment. TNF treatment does activate NF-*κ*B in tumor cells, as judged by increased levels of the phosphorylated form of the p65 subunit (NF-κBp65^P-Ser276^) and this was reduced by PDTC treatment. PDTC did not affect TNF induction of RIPK1 and RIPK3 expression in TECs (Supplementary Figures 7A and 7B). NFκB activation often protects cells from TNF-induced apoptosis through the induction of c-FLIP, a protein that competes with caspase 8 for binding to FADD, a key step for autocatalytic caspase 8 activation. We observed TNF-induced activation of caspase 8^p18^ primarily in infiltrating leukocytes and only a weak infrequent signal in tumor cells. Interestingly, c-FLIP was present at high levels in untreated tumor cells and its expression levels are unaltered by TNF treatment (Supplementary Figure 8). These findings suggest that induction of RIPK1 and RIPK3 by TNF in TECs is independent of NF*κ*B and that constitutive overexpression of c-FLIP in the tumor cells^35, 36^ may confer resistance against caspase 8-induced apoptosis in RCC resulting in dominance of necroptotic cell death.

### R1TNF activates signaling pathways associated with necroptosis in RCC

**Figure 3:**
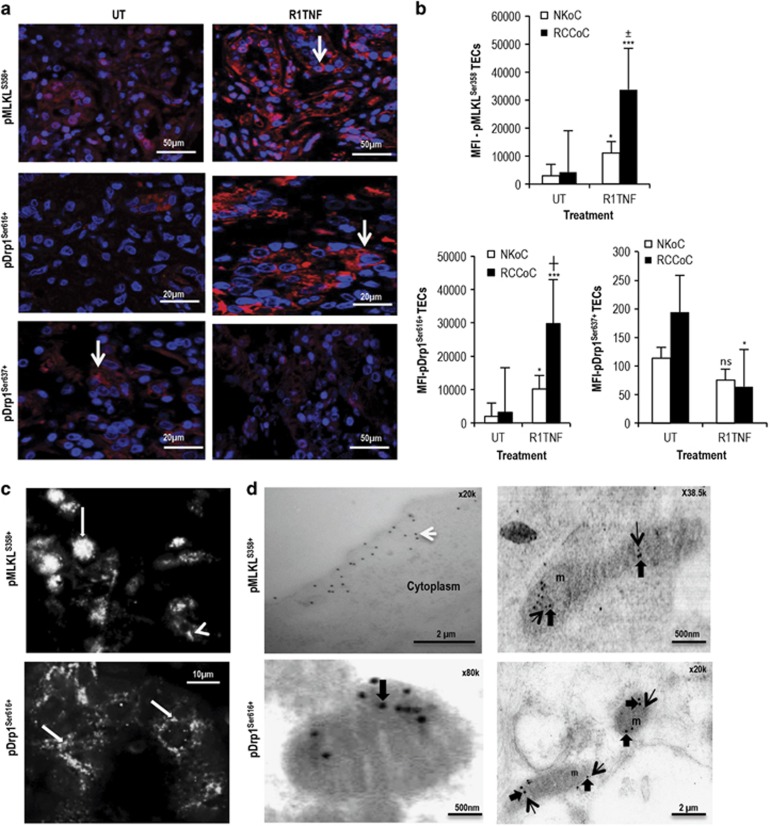
Effect of R1TNF on pMLKL^Ser358^, pDrp1^Ser616^ and pDrp1^Ser637^ expression in organ cultures of RCC grade 1 and adjacent non-tumor kidney (NK). (**a**) R1TNF induced a marked expression of pMLKL^S358^ and pDrp1^Ser616^ but a reduced signal for pDrp1^Ser637^ compared with untreated controls (UT), with signal mainly confined to mTECs (arrows). (**b**) Quantification of the phosphorylated proteins in normal and mTECs presented as mean fluorescent intensity (MFI) shows a statistical significant difference between cultures. ^***^P<0.001 *versus* UT, *P<0.05 *versus* UT; ^±^*P*<0.05 *versus* R1TNF (NKoC); ^┼^*P*<0.001 *versus* R1TNF (NKoC); ns, not significant. (**c**) pMLKL^Ser358^ is seen within cytoplasm (arrowhead) and in some nuclei (arrow) (*upper panel*), while pDrp1^Ser616^ show cytoplasmic granular pattern (arrows) (*lower panel*). (**d**) Immunogold electron microscopy revealed pMLKL^Ser358^ (15 nm particles) on the cell surface (black arrows), within cytoplasm (white arrow) and in mitochondria (m), while pDrp1^Ser616^ (5 nm particles, black arrow) was mainly confined to mitochondria (m) (open arrows). Bars=mean±S.E.M.; *n*=3 independent experiments from six separate organ culture experiments with similar results

To determine whether downstream targets of RIPK3 are present and activated in organ cultures of RCC and NK in response to TNF, we examined the presence and phosphorylation of MLKL and Drp1. In UT controls, both RCC (Supplementary Figure 9) and NK (data not shown) showed strong expression of MLKL and Drp1 in mTECs and normal TECs as well as in infiltrating MNCs and some vascular endothelial cells. However, compared with UT controls, R1TNF increased the expression of phosphorylated MLKL at Ser358 (MLKL^Ser358^) and phosphorylated Drp1 at Ser616 (pDrp1^Ser616^) in RCC-mTECs by ~5–10-fold, and reduced pDrp1 at Ser637 (pDrp1^Ser637^) by ~2-fold (Figure 3a, quantified in Figure 3b). Cultures of NK showed a reduced response with about a ~2–3-fold rise in pMLKL^ser358^ and pDrp1^ser616^ and a similar reduction in pDrp1^ser637^ (quantified in Figure 3b). pMLKL^Ser358^ was mainly diffusely cytoplasmic and nuclear, while pDrp1^ser616^ showed a cytoplasmic granular pattern (Figure 3c), consistent with Drp1 binding to mitochondria. Binding specificity for antibodies to MLKL, Drp1, pMLKL^Ser358^, pDrp1^Ser637^ and pDrp1^Ser616^ was confirmed by incubation with a corresponding blocking peptide and by gene knockdown studies in human HEK293 cells (Supplementary Figures 10A and 10B). Indeed, immunogold electron microscopy of R1TNF-treated cultures showed pMLKL^Ser358^ on the cell surface, within the cytoplasm and in some mitochondria, while pDrp1^ser616^ was mainly localized to mitochondria (Figure 3d). wtTNF induced comparable levels of signal for both phosphorylated proteins while the response with R2TNF in RCC was similar to that of UT controls (Supplementary Figure 11).

**Figure 4:**
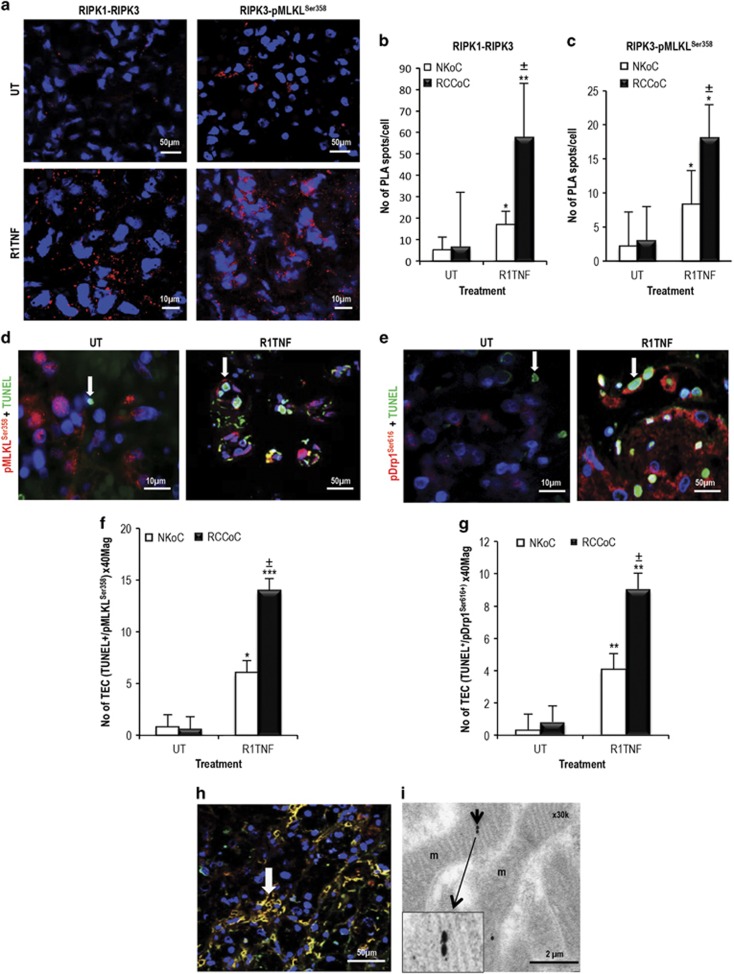
PLA of organ culture of RCC grade 1. (**a**) In comparison with untreated controls (UT), R1TNF induced a strong interaction of RIPK1-RIPK3 and RIPK3-pMLKL^Ser358^ appearing as strong red fluorescence spots mainly within the cytoplasm of mTECs. Each individual interacting protein pair observed as a red spot by confocal microscopy is expressed as the number of signals/cell (PLA spots/cell). (**b** and **c**) Quantification of PLA spots in TECs in the two study groups; RCC (RCCoC) and normal kidney (NKoC) show a statistically significant difference, more pronounced in RCCoC. ^**^P<0.01 *versus* UT, *P<0.05 *versus* UT, ^±^*P*<0.05 *versus* R1TNF (NKoC). (**d** and **e**) Representative confocal images of pMLKL^Ser358^ or pDrp1^Ser637^ and TUNEL in organ cultures of RCC grade 1. Compared with UT cultures, R1TNF induced an increase in the level of ^TUNEL+^mTECs (*green*) associated with pMLKL^Ser358^ and pDrp1^Ser616^ (*red*) expression (arrows). (**f** and **g**) Quantification of ^TUNEL+^mTECs/pMLKL^Ser358+^ and ^TUNEL+^mTECs/pDrp1^Ser616+^ shows statistical significant differences between cultures. ^***^P<0.0001 *versus* UT, ^**^P<0.001 *versus* UT, *P<0.01 *versus* UT, ^±^*P*<0.05 *versus* R1TNF (NKoC). (**h**) Combined immunofluorescence of R1TNF-treated cultures shows co-localization of pMLKL^Ser358^ and pDrp1^Ser616^ in mTECs (arrow). (**i**) Immunogold electron microscopy demonstrate close proximity of gold particles for pMLKL^Ser358^ (5 nm) and pDrp1^Ser616^ (15 nm) in mitochondria (m) (*inset zoomed × 2.5*). Bars=mean±S.E.M.; images are representative of *n*=3 independent experiments from six separate organ culture experiments with similar results

To evaluate whether TNF mediates an interaction between RIPK1 and RIPK3 and RIPK3 and pMLKL^ser358^, we performed a proximity ligation assay (PLA) in organ cultures of RCC and NK (Figure 4a, quantified in Figures 4b and c). R1TNF enhanced the RIPK1-RIPK3 interaction by about ~10-fold, evidenced by numerous strong red fluorescent spots within the cytoplasm of mTECs in RCC (~60 PLA spots/cell) compared with UT controls (~6 PLA spots/cell), whereas the interaction of RIPK1-RIPK3 induced by R1TNF in NK was only about 3-fold (quantified in Figure 4b). In addition, a fluorescent signal indicating a RIPK3-pMLKL^ser358^ interaction increased by ~5-fold in mTECs in R1TNF-treated RCC cultures (~16 PLA spots/cell) compared with UT (~3 PLA spots/cell), whereas the increase in NK was about ~2.5-fold (quantified in Figure 4c). Data from wtTNF gave similar results as R1TNF, while R2TNF showed comparable data as UT controls (Supplementary Figures 12A-C).

To demonstrate that cells with higher pMLKL^Ser358^, pDrp1^Ser616^ and lower pDrp1^Ser637^ are the cells that are prone to die, we subjected all cultures to TUNEL in combination with IF for pMLKL^Ser358^, pDrp1^ser616^ and pDrp1^Ser637^. We found a significant >60% association between pMLKL^ser358^ and ^TUNEL+^mTECs in R1TNF-treated cultures of RCC compared with UT controls (<2%), which showed fewer numbers of ^TUNEL+^mTECs and no association with pMLKL^Ser358^ (Figure 4d, quantified in Figure 4f). An association of >40% between pDrp1^Ser616^ and ^TUNEL+^mTECs was also detected in R1TNF-treated RCC organ cultures (Figure 4e, quantified in Figure 4g, Table 1). wtTNF showed similar findings to R1TNF. Although R2TNF also increased pMLKL^Ser358^ in some ^TUNEL+^mTECs, it was not significant compared with UT controls (Supplementary Figure 13A, quantified in Supplementary Figures 13B and 13C). To determine whether pDrp1^Ser616^ co-localizes with pMLKL^Ser358^ in mTECs, we co-stained for pDrp1^Ser616^ and pMLKL^Ser358^ in organ cultures of RCC cells treated with R1TNF and demonstrated a moderate association (>20%) (Figure 4h) confirmed by immunogold EM showing staining in mitochondria (Figure 4i). Similar, but much reduced, effects of R1TNF on necrotic cell death and associated signaling pathways were evident in TECs in NK (quantified in Supplementary Figures 13B-C).

### Inhibitors of apoptosis and necroptosis differentially prevent TNF-mediated cell death in RCC organ cultures

**Figure 5:**
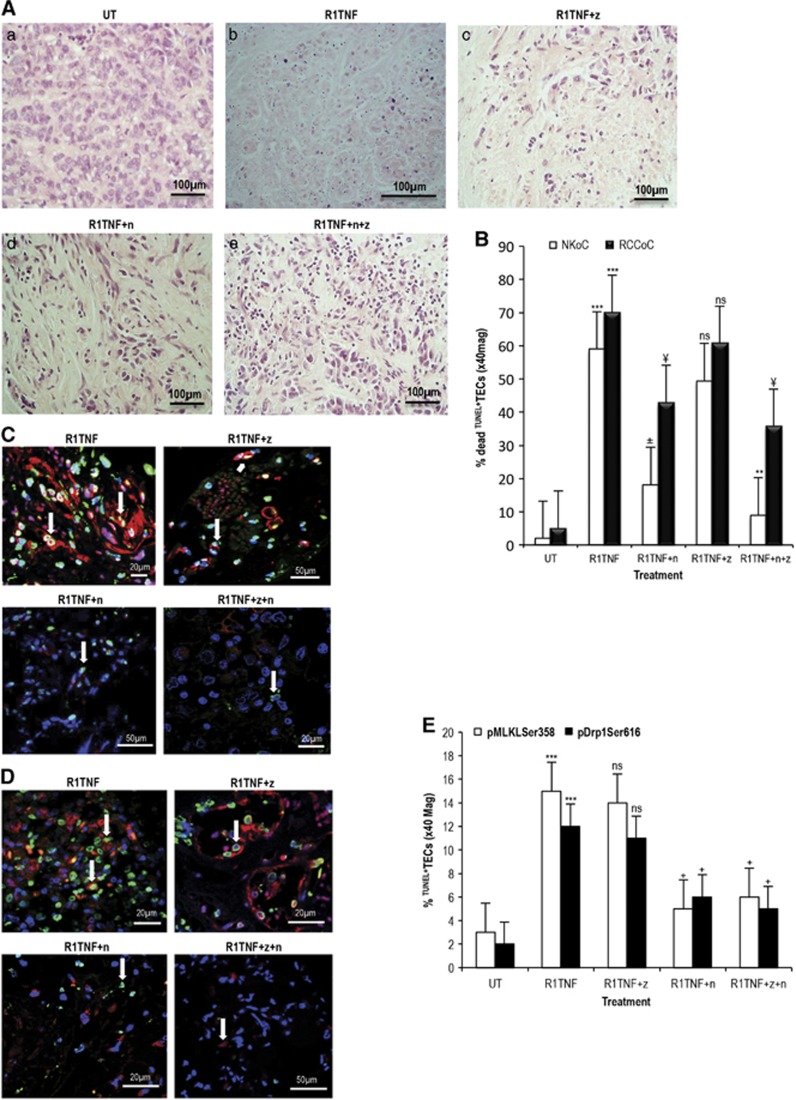
(**A**) Representative hematoxylin and eosin-stained sections demonstrate morphological features of necrosis in organ cultures of grade 1 ccRCC treated with or without necrostatin-1 or/and zVAD.fmk followed by R1TNF. R1TNF induced increased cell death compared with untreated (UT) cultures (a and b). Treatment with zVAD.fmk (z) (c) resulted in partial inhibition of cell death, further rescued by necrostatin-1 (n) (d), even more so by a combination of z and n (e). (**B**) Quantification of parallel sections stained with TUNEL show the precentage of dead tubular epithelial cells (TECs) in 10 random high power fields at × 40 magnification. ^***^P<0.0001 *versus* UT; ^**^P<0.001 *versus* R1TNF+n (NKoC), ^±^*P*<0.01 *versus* R1TNF (NKoC), ^¥^*P*<0.05 *versus* R1TNF (RCCoC); ns, not significant. (**C** and **D**) R1TNF induced increased level of ^TUNEL+^mTECs (*green*), also expressing pMLKL^Ser358^ or pDrp1^Ser616^ (arrows) (*red*). Treatment with zVAD.fmk (z) partially reduced the number of ^TUNEL+^mTECs (arrows) but did not have any effect on pMLKL^Ser358^ or pDrp1^Ser616^ expression. In contrast, treatment with necrostatin-1 (n) resulted in a significant reduction in the number of ^TUNEL+^mTECs, associated with a diminished level of pMLKL^Ser358^ or pDrp1^Ser616^ comparable with cultures treated with a combination of zVAD.fmk and necrostatin-1 (z+n) (arrows). (**E**) Quantification of the percentage of ^TUNEL+^mTECs in combination with pMLKL^Ser358^ or pDrp1^Ser616^ in 10 random high power fields at × 40 magnification; ^***^P<0.001 *versus* UT; ^+^*P*<0.05 *versus* R1TNF; ns, not significant. Bars=mean±S.E.M. Images are representative of *n*=3 independent experiments from six separate organ culture experiments with similar results

To further define the TNF-mediated cell death pathway in RCC, we utilized specific inhibitors. Cultures were treated with zVAD.fmk, a pan-caspase inhibitor,^37^ or necrostatin-1 (Nec-1), an inhibitor of RIPK1 kinase activity, and cell death was quantified on TUNEL stained sections.^8^ zVAD.fmk inhibited cell death by ~10%, whereas Nec-1 by about ~40–60% (Figure 5A, quantified in Figure 5B). The combination of Nec-1 and zVAD.fmk was additive, indicating that apoptosis and necrosis occur in separate cells (Table 2). R2TNF also induced death of mTECs but to a lesser extent than R1TNF, with no significant inhibition by zVAD.fmk, and a small but significant inhibition with Nec-1. Similar but less marked effects were observed in NK (Table 2).

Next, we determined whether inhibition of cell death by zVAD.fmk or Nec-1 or their combination correlates with R1TNF-mediated phosphorylation of MLKL^Ser358^ and Drp1^Ser616^. In the presence of zVAD.fmk, there was no obvious reduction in the expression of the phosphorylated proteins although there was a mild decrease in the number of ^TUNEL+^mTECs associated with pMLKL^Ser358^ and pDrp1^Ser616^ (~14%) (Figures 5C and D, quantified in Figure 5E). In contrast, the reduction in ^TUNEL+^mTECs by Nec-1 was associated with a diminished level of both phosphorylated proteins (~3-fold), comparable with that found in cultures treated with a combination of zVAD.fmk and Nec-1. Thus, the decrease in the level of MLKL^Ser358^ and Drp1^Ser616^ are strongly correlated with the decrease in ^TUNEL+^mTECs only after treatment of Nec-1 and not zVAD fmk, indicating that the necrosis is not secondary to apoptosis.

**Figure 6:**
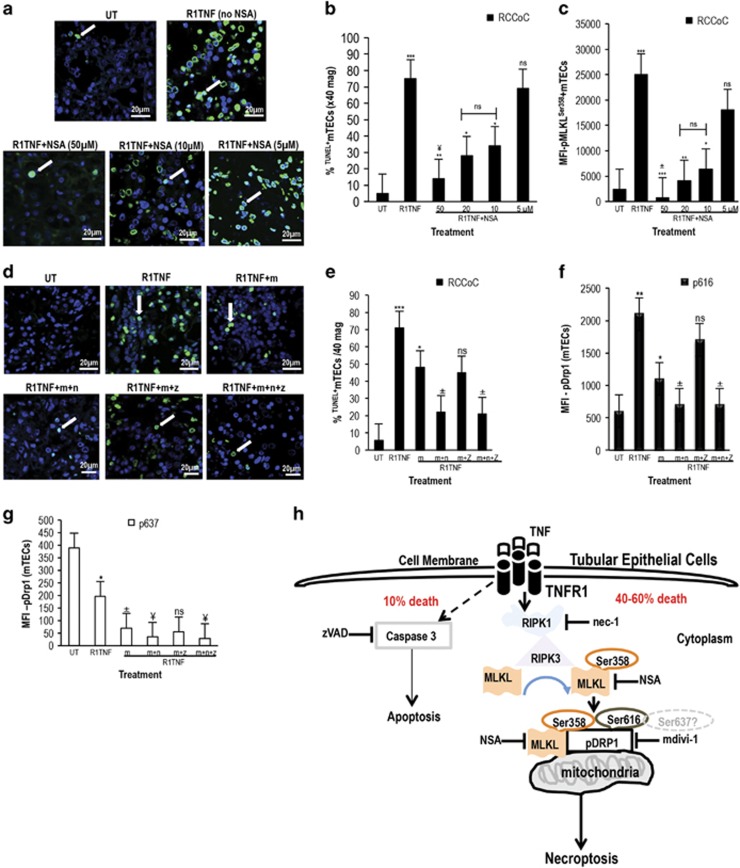
(**a**) Effect of necrosulfonamide (NSA) and mdivi-1 (m) in grade 1 RCC organ cultures treated with R1TNF. R1TNF alone without NSA (no NSA) induced increased levels of ^TUNEL+^mTECs, which were significantly reduced with 50 *μ*M NSA, and to a lesser extent, with 10 *μ*M or 20 *μ*M NSA (arrows). Cultures pretreated with 5 *μ*M NSA showed comparable levels of ^TUNEL+^mTECs as untreated cultures (UT). (**b**) The percentage of ^TUNEL+^mTECs and (**c**) pMLKL^Ser358^ expression presented as mean fluorescent intensity (MFI) in similar cultures. ^***^P<0.0001 *versus* UT,^**^P<0.001 *versus* R1TNF; *P<0.05 *versus* R1TNF; ^¥^*P*<0.05 *versus* R1TNF (+20 or 10*μ*M); ^±^*P*<0.001 *versus* R1TNF (+20 or 10 *μ*M); ns, not significant. (**d**) In comparison with UT, which show a rare ^TUNEL+^mTECs, R1TNF alone (without m) induced increased levels of ^TUNEL+^mTECs, significantly reduced by m (10 *μ*M) with no effect by zVAD.fmk (m+z) but a marked reduction by nec-1 (m+n) comparable with cultures pretreated with a combination of zVAD.fmk and nec-1 (m+n+z). (**e**) The percentage of ^TUNEL+^mTECs and (**f** and **g**) the mean fluorescence intensity (MFI) for pDrp1^Ser616^ and pDrp1^Ser637^ in similar cultures. ^***^P<0.0001 *versus* UT (**e**), ^**^P<0.01 *versus* UT (**f**), *P<0.05 *versus* R1TNF, ^±^ *P*<0.05 *versus* R1TNF+m. (**g**) *P<0.05 *versus* UT, ^±^*P*<0.5 *versus* R1TNF, ^¥^*P*<0.05 *versus* R1TNF+m; ns, not significant. Bars=mean±S.E.M.; *n*=3 independent experiments from six separate organ culture experiments with similar results. (**h**) Schematic diagram of the consequences of R1TNF-mediated necroptosis in mTEC in RCC; ligation of TNFR1 results in the recruitment of RIPK1, facilitating its interaction with RIPK3, which in turn recruits and phosphorylates MLKL at Ser358 and Drp1 at Ser616 thus causing their co-localization with the mitochondria. A separate process causes a reduction in pDrp1 at ser637. Nec-1 inhibits RIPK1, and NSA inhibits MLKL and mdivi-1 inhibits Drp1 inhibiting cell death

To test whether pMLKL^Ser358^ has a functional role in TNF-mediated death of mTECs in RCC, we applied NSA, which blocks programmed necrosis by specifically targeting MLKL.^15^ The number of R1TNF-induced ^TUNEL+^mTECs was reduced by NSA treatment in a concentration-dependent manner (Figure 6a, quantified in Figure 6b) (UT~5%, R1TNF~75% R1TNF+50 *μ*M NSA~15% R1TNF+20 *μ*M NSA~30% R1TNF+10 *μ*M NSA~35% R1TNF+5 *μ*M NSA~70%). A similar but reduced effect of NSA was evident in NK organ cultures (data not shown). NSA inhibition of mTEC death correlated with a concentration-dependent reduction of pMLKL^ser358^ expression (Figure 6c). wtTNF (without NSA) induced comparable levels of death in mTECs as R1TNF with similar inhibitory effects of NSA (data not shown). R2TNF induced an insignificant increase in mTEC death as compared with UT controls.

To investigate the importance of pDrp1 in TNF-mediated death of mTECs in RCC, we used mdivi-1, a small molecule inhibitor of Drp1.^38, 39, 40^ Treatment of organ cultures with mdivi-1 inhibited wtTNF- and R1TNF-mediated alterations in the phosphorylation status of Drp1, and reduced increases in ^TUNEL+^mTECs, which was further reduced by treatment with a combination of mdivi-1 and Nec-1 (Figure 6d, quantified in Figure 6e) (UT~5% R1TNF~72% R1TNF+m~48% R1TNF+m+n~22% R1TNF+m+z~45% R1TNF+m+n+z~21%). mdivi-1 inhibition of mTEC death correlated with a significant reduction of pDry1^Ser616^ and pDrp1^Ser637^ (Figures 6f and 6g). Similar effects of wtTNF and R1TNF-induced ^TUNEL+^mTECs were observed in NK organ cultures but to a lesser extent than in RCC organ cultures (data not shown).

## Discussion

TNF was initially described as a factor present in the blood of Bacillus Calmette–Guerin-infected mice treated with endotoxin^41, 42^ where it was shown to be an important mediator of the priming phase of a Shwartzman-like reaction involving neutrophil activation and intravascular coagulation.^43^ Vascularized tumors showed evidence of hemorrhagic necrosis, hence the name TNF. Various *in vitro* studies later reported that in most cultured tumor cells, TNF caused apoptotic cell death,^44, 45^ although there were some exceptions that appeared to show death with features of necrosis (e.g., L929 cells).^46^ The relationship between tumor cell apoptosis *in vitro* and necrosis *in vivo* was not clear. However, in the last few years, it has been appreciated that features of necrosis can also be induced in individual cells in culture^6, 7, 18, 23, 47^ and TNF-induced necrosis, like apoptosis, is thought to be predominantly mediated by TNFR1 signaling.^2^ Which form of programmed death predominates *in situ* is open to debate. Our study is unique in that it is based on an organ culture system so that cells are studied *in situ* but death cannot be attributed to Shwartzman-type phenomena. Thus, necroptosis and indirectly caused necrosis can be separated and this may be the first example in which this approach has been used. *In vitro* studies have utilized antibodies to localize distinct proteins that participate in TNF-induced necroptosis and changes in their phosphorylation status.^23, 48^ We have taken advantage of the pharmacological inhibitors of these proteins to monitor cell death combined with immunohistochemistry at the light and electron microscopic level to determine the effect(s) of TNF in RCC and NK. Consistent with our earlier report,^27^ we show morphological evidence of death in mTECs of RCC cultures treated with wtTNF and R1TNF, and to a minor extent TNFR2. This was associated with upregulation of RIPK1 and RIPK3 mRNA and protein expression, kinases involved in TNF-mediated necroptosis.^2, 6, 10, 33, 49, 50, 51, 52, 53^ We further show a significantly elevated RIPK1 and RIPK3 protein expression in mTECs of high grade (3/4) as compared with low-grade tumors (1/2). Upregulation of RIPK1 has been reported in lung cancer and glioblastoma tissues,^54, 55^ but not in colon cancer,^52^ suggesting that distinct mechanisms of cell killing may exist in different tumor types. Inhibition of NF*κ*B by PDTC did not abolish TNF-induced RIPK1 and RIPK3 in our organ cultures suggesting that activation of these kinases occurs independent of NF*κ*B, in line with O'Donnell *et al.,*^56^ who reported that K63-linked ubiquitination of RIPK1 occurs early after TNFR1 stimulation and does not require NF*κ*B transcription. In RCC, it is possible that the higher expression of RIPK1/3 renders them more prone to TNF-mediated necroptosis, suggesting that differential treatments may be devised to augment chemotherapeutic death in RCC while sparing normal surrounding tissue. Our observation of the small amount of TNFR2-mediated death of mTECs is consistent with a proposal by Chan *et al.*^11^ that TNFR2 signaling does not directly engage the cell death machinery, but rather enhances TNF-induced necroptosis indirectly via TNFR1 by recruitment of RIPK1.^11^ If TNFR1 is activated by TNFR2 in RCC independently of TNFR1 occupancy, it is a very minor response.

In the next steps, we underpinned the necroptotic pathway by demonstrating a direct interaction between RIPK1 and RIPK3, between RIPK3 and MLKL, and the participation of MLKL and Drp1 in the necrotic process, as outlined in the scheme shown in Figure 6h. Previous work has shown that RIPK3 is recruited to RIPK1 through their RHIM domains upon execution of necrosis.^7, 11, 15, 52^ The pharmacological inhibition of RIPK1 abolishes the recruitment of RIPK3 and thereby inhibits RIPK3 activation, suggesting that RIPK1 is upstream of RIPK3.^7^ However, a recent report has suggested that RIPK3 oligomerization is sufficient to induce necroptosis, independent of the RHIM domain.^57^ Nevertheless, RIPK3 is now known to be the molecular switch for necroptosis,^58, 59, 60, 61^ and its expression renders cells permissive to necroptosis upon TNF treatment^7, 10^ via MLKL^31, 62^ and Drp1 recruitment and phosphorylation. Using the PLA assay,^63^ we show that stimulation of RCC organ cultures with wtTNF or R1TNF induced RIPK1-RIPK3 interaction in mTECs, consistent with previous reports.^6, 7, 10^ Furthermore, we found that R1TNF induced an association between RIPK3 and pMLKL^Ser358^ and that R1TNF increased pMLKL^Ser358^ and pDrp1^Ser616^ and reduced Drp1^ser637^ in mTECs.

The increase in pMLK^Ser358^ and pDrp1^Ser616^ in TECs was associated with ^TUNEL+^mTECs, further supporting a role for these phosphorylated proteins in necroptosis. Interestingly, immunogold electron microscopy analysis of R1TNF-treated cultures of RCC demonstrated the presence of pMLKL^Ser358^ in some mitochondria and on the plasma membrane of mTECs. This result is consistent with that of Cai *et al.*,^64^ who reported translocation of RIPK3-dependent phosphorylated MLKL homotrimers to the cell plasma membrane, a process which leads to cell rupture. This finding is also supportive of a recent report by Rodriguez *et al.*^20^ Our immunofluorescence studies of wtTNF and R1TNF-treated cultures of RCC also demonstrated induction of pDrp1^Ser616^ and its co-localization with pMLKL^Ser358^ in mTECs, and immunogold EM analysis further confirmed association of pMLKL^Ser358^ and pDRP1^Ser616^ in the mitochondria of mTECs, implying that mitochondria may be a point of aggregation for execution of necrosis by these death-inducing components.^65^

Drp1 is regulated by posttranslational modifications such as phosphorylation.^66, 67^ Drp1 phosphorylation at Ser616 has been linked to cancer invasion and growth and its increased expression has been reported in human lung cancer.^68, 69^ In our hands, increased pDrp1^Ser616^ was associated with cell death; perhaps, its effects are context-dependent, so it couples to necroptosis when associated and co-localized with pMLKL^Ser358^ but not in other environments. Differing extents of Drp1 and mitochondrial involvement in regulation of necroptosis have been reported, which most likely depend on the model studied. In the human organ culture model used here, there is a heterogeneous cell population, so factors that exert a spectrum of bioactivities may influence cell death regulation differently to experiments in a pure cell line. Our observation of rescued cell death mediated by TNF using mdivi-1, a selective inhibitor of Drp1, its correlation with phosphorylation of Drp1 at Ser616 and its co-localization with mitochondria strongly implicate them as likely targets in TNF-mediated necroptosis in RCC, though we have not proven this conclusively. In contrast to pDrp1^Ser616^, wtTNF and R1TNF reduced pDrp1^ser637^ expression in mTECs. Loss of this site is linked to Drp1 activation, a process thought to be regulated by mitochondrial protein phosphatase PGAM5.^15, 23^ However, the requirement for PGAM5 in necroptosis induction has been challenged by others.^70^ Interestingly, necroptosis pathway inhibitors did not reverse the reduction in pDrp1^Ser637^ induced by R1TNF, so its relationship to the necroptosis pathway is unclear. Further work will be required to elucidate its role in TNF signaling and whether PGAM5 is important for mediating TNF-induced necroptosis in RCC. Collectively, our data strongly suggest that TNF-induced necroptosis in mTECs in RCC occurs via altered phosphorylation of MLKL/Drp1 and that this process may involve mitochondrial localization and possibly the plasma membrane. However, further studies will be needed to clarify the causal connections.^48^

Finally, we analyzed how these components contribute to cell death using small molecule inhibitors. It is well established that secondary necrosis can occur downstream of apoptosis, which is different from necroptosis.^29, 30^ To test whether this explains the necrosis in our cultures, we used zVAD.fmk, a pan-caspase inhibitor.^10, 32, 50, 58^ We show that treatment with zVAD.fmk resulted in only a minor inhibition of TNFR1-mediated cell death in mTECs consistent with our previous report that R1TNF induces expression of cleaved active caspase 3^p175^ in some mTECs.^34^ However, Nec-1, an allosteric RIPK1 inhibitor was highly effective at preventing cell death consistent with previous reports,^50, 51^ and the combined addition of zVAD.fmk and Nec-1 appeared to have an additive effect. These data imply dominance of a caspase-independent mode of cell death in mTECs, consistent with other studies.^71^

To further characterize the specific roles of MLKL and Drp1 in TNF-induced necroptosis in organ cultures of RCC and NK, we utilized the inhibitors NSA (inhibitor of MLKL) and mdivi-1 (a selective inhibitor of Drp1). We found that NSA reduced the number of ^TUNEL+^mTECs back to untreated levels, while mdivi-1 was less effective at the concentration used. NSA- and mdivi-1-mediated death inhibition was associated with a significant reduction in expression of the relevant phosphorylated proteins; pMLKL^Ser358^ by NSA and pDrp1^Ser616^ by mdivi-1. NSA also attenuated phosphorylation of Drp1^Ser616^ (data not shown), consistent with a previous report.^23^ mdivi-1 is a quinazolinone derivative, attenuating Drp1 self-assembly, thereby causing the inhibition of mitochondrial fission. Owing to its potential in preventing mitochondrial fragmentation, mdivi-1 has shown protective efficacy in a number of disease models, including acute kidney injury, heart ischemia/reperfusion injury and Parkinson's disease.^72^ Our findings are consistent with earlier reports that demonstrate a protective effect of mdivi-1 by attenuating R1TNF-induced necroptotic death.^72, 73^ The pathway we uncovered of R1TNF-mediated necroptosis in mTECs in RCC is schematized in Figure 6h.

In summary, our data provide new evidence demonstrating that TNF is an inducer of necroptosis in mTECs in organ culture via TNFR1, that necroptosis is the predominant form of cell death and that TNF-regulated necrosis occurs through a RIPK1/RIPK3/MLKL/Drp1 axis. Given that our system is closer to the situation *in vivo*, our findings support the development of a therapeutic strategy targeting non-apoptotic cell death pathways in RCC especially if they are resistant to pro-apoptotic treatment.

## Materials and Methods

### Reagents/antibodies

The antibodies used were as follows: anti-RIPK1 (cat~NBP1-77077) (Novus Biologicals, Oxford, UK), anti-RIPK1 (cat~3493S) (New England Biolabs, Hitchin, UK), anti-RIPK1 (cat~ab56815), anti-RIPK3 (cat~ab16090), anti-RIPK3 (cat~ab56164) (Abcam, Cambridge, UK). Anti-RIPK3 (cat~GTX107574, GenTex, Irvine, CA, USA), anti-RIPK3 (cs-13526S), anti-MLKL and anti-phosphorylated MLKL^Ser358^ (phospho-Ser358) (cat~17-10400) and anti-mitochondria (MAB1273, Millipore, Watford, UK); anti-phosphorylated MLKL^Ser358^ (cat~ab187091), anti-c-FLIP (cat~ab6144) and anti-Caspase-8^p18^ (cat~ab25901) are from Abcam, anti-NF*κ*Bp65^p-ser276^ (cat~3037S), anti-pDrp1^Ser616^ (cat~cs-3455S), anti-pDrp1^Ser637^ (cat~cs-4867S) are from New England Biolabs. Rabbit anti-cytokeratin (CK) (cat~sc15367) is from Insight Biotechnology Ltd, Wembley, UK, and mouse anti-CK (cat~VP-C420) is from Vector Laboratories, Peterborough, UK. Blocking peptides for RIPK1 (cat~NBP1-77077PEP) are from Novus Biologicals and for RIPK3 (~ab178834) and pMLKL^Ser358^ (ab206929) are from Abcam. zVAD.fmk (pan-caspase inhibitor N-benzyloxycarbonyl-Val-Ala-Asp-fluoromethylketone; cat~G7231) is from Promega, Madison, WI, USA; PDTC, 3,3′-diaminobenzidine substrate (DAB), Necrostatin-1 (Nec-1, cat~N9037) and mdivi-1 (3-(2, 4-dichloro-5-methoxyphenyl)-2-sulfanyl-4(3H)-quinazolinone) are from Sigma-Aldrich (Gillingham, UK). TUNEL-*In Situ* Cell Death Detection Kit (cat~11767291910) is from Roche Diagnostics, West Sussex, UK. Five nanometer and 15 nm-conjugated gold particles are from British Biocell (Cardiff, UK); Hoechst 33342 is from Thermo Fisher Scientific (Paisley, UK); wild-type TNF (wtTNF) (cat~210-TA-020; R&D systems, Oxford, UK) and TNFR-specific muteins (R1TNF and R2TNF) were a generous gift from Professor Peter Vandenabeele (Gwent, Belgium).

### RCC and NK organ cultures

All experiments using human tissue were performed with the written informed consent of patients and the approval of the local Ethical Committee and Addenbrooke's Hospital Tissue Bank. RCC tissue grade 1 and adjacent non-tumor kidney tissue (NK) (*n*=6) from radical nephrectomies were randomly dissected and processed for organ culture as previously reported.^74^ Briefly, duplicate <1 mm^3^ fragments were placed in M199 medium in 96-well plates and either left in medium alone (untreated; UT) or pretreated with wtTNF (10 ng/ml) or R1TNF or R2TNF (1 *μ*g/ml) for 3 h at 37 °C. Parallel cultures were pretreated with zVAD.fmk (20 *μ*M)^50, 51, 58, 75^ or Nec-1 (30 *μ*M)^51, 76, 77^ or with a mixture of zVAD.fmk and Nec-1 for 1h prior to wtTNF or R1TNF or R2TNF. In addition, some cultures were pretreated for 1h with different dose of necrosulfoximide (NSA; inhibitor of pMLKL) (5, 10, 20, 50 *μ*M) or mdivi-1 (10 *μ*M; mitochondrial division inhibitor-1 targets pDrp1) before TNF or R1TNF or R2TNF with Nec-1 and/or zVAD.fmk. Additional, duplicate samples were either treated with 50 *μ*g/ml (or 300 *μ*M) PDTC^34^ or left untreated for 30 min before TNF treatment. All cultures were fixed in 4% paraformaldehyde and wax sections stained with hematoxylin and eosin for morphological studies, immunofluorescence, immunohistochemistry, TUNEL and *in situ* hybridization. Images of hematoxylin and eosin sections were captured using a Leitz Laborlux 12 Microscope with Infinity 2 camera (Lumenera Corporation, Ontario, Canada).

### Immunohistochemical and immunofluorescence analyses (IHC and IF)

RCC, NK and corresponding organ cultures were subjected to IHC and IF as previously described.^74^ Briefly, some sections were incubated with anti-RIPK1, -RIPK3, -pMLKL^Ser358^, -pDrp1^Ser616^, pDrp1^Ser637^ and pan-cytokeratin and some sections immunostained for pMLKL^Ser358^ and -pDrp1^Ser616^ or pDrp1^Ser637^ using Zenon Fab reagents,^78^ followed by incubation with flourochrome-conjugated secondary antibody (Northern Light^498^ or ^557^) (R&D Systems) plus Hoechst 333342. For IHC, sections were pretreated with 30% H_2_O_2_ before incubation with primary antibodies, followed by a peroxidase-conjugated secondary antibody and DAB as chromogen and counterstained with hematoxylin. Parallel cultures treated with or without PDTC were also examined for expression of NF*κ*Bp65^p-ser276^, c-FLIP and active Caspase 8^p18^. Negative controls included pre-adsorption of primary antibodies with blocking peptides overnight at 4 °C prior to immunostaining and also replacement of the primary antibody with an isotype-specific sera. To further confirm specificity of the antibodies to RIPK1, RIPK3, MLKL and DRP1 used in this study, we carried out gene knockdown experiments on human HEK293 cells using siRNA against each target protein (as these cells show high transfection efficiency). For this, cells were reverse-transfected with 100 nM of the appropriate siRNA (GE Healthcare UK Ltd, Buckinghampshire, UK) in 8-well slide chambers using the TurboFect transfection reagent (Thermo Fisher Scientific), according to the manufacturer's protocol. After 48 h of transfection, the cells were treated with zVAD.fmk (25 *μ*M) and the Smac-mimetic LCL-61 (100 *μ*M) (Selleckchem, Houston, TX, USA) for 30 min prior to treatment with TNF (10 ng/ml) for 4h and then subjected to IF. siRNAs used include Accell Human Control siRNA non-targeting pool Cat~D-001910-10-20; Accell Human RIPK1 siRNA SMARTpool Cat~E-004445-00-000, Accell Human RIPK3 siRNA SMARTpool Cat~E-003534-00-0005, Accell Human MLKL siRNA SMARTpool Cat~ E-005326-00-0005, Accell Human DRP1 siRNA SMRTpool Cat~E-012092-00-0005 (GE Healthcare Ltd).

Photomicrographs were captured using a on a SPE-confocal laser scanning microscopy (Leica Microsystems Ltd, Milton Keynes, UK) and IF images were captured on a confocal laser scanning microscopy. The mean fluorescence intensity was quantified in Image J. All data were transferred to GraphPad Prism 5.0 for statistical analysis.

### *In situ* PLA

The principle of the *in situ* PLA was reported previously.^63^ Briefly, when two proteins are closer than 40 nm, signals can be detected. *In situ* PLA assay was performed using the Duolink *In Situ* Detection Regents Red (Cat~DUO92008; Sigma-Aldrich) according to the manufacturer's instructions. For details, refer to Supplementary Methods. The number of *in situ* fluorescent PLA signals (red spots) was counted in mTECs in 10 random fields of view at × 40 magnification. The number of signals per cell in the negative controls incubated with only one of the primary antibodies (RIPK1 and RIPK3) was also counted and the increase in signal calculated as the ratio of the number of signals per cell in the sample divided by the sum of the signals in the negative controls, as previously reported.^79^ Images were acquired on a confocal laser scanning microscopy and prepared using Photoshop CS6 software.

### *In situ* hybridization (ISH)

As previously described,^74^ paraffin sections of RCC and NK organ cultures were hybridized overnight at 37 ^°^C with single-stranded anti-sense DNA oligonucleotide probes 5′-end labeled with digoxigenin specific for human RIPK1 (NM_003804) or human RIPK3 (NM_006871) (Eurofins Genomics, Ebersberg, Germany) followed by anti-digoxigenin-11-dUTP-conjugated-alkaline phosphatase (AP) (Roche). Gene expression was visualized as described in Supplementary Methods.

### Terminal deoxynucleotidyl transferase (TdT)-mediated-digoxigenin-11-dUTP nick end labeling (TUNEL)

TUNEL was used to detect DNA fragmentation in RCC and NK organ cultures treated with wtTNF, R1TNF and R2TNF as previously described.^74^ The number of TUNEL^+^ dead TECs per total number of viable cells were counted in 10 high power fields of view at × 40 magnification and scored by two observers blinded to the treatments and the data presented as percentage of dead TECs. For details of TUNEL assay, see Supplementary Methods.

### Immunoblotting

RCC and NK tissue were processed for immunoblotting as previously described.^27^ Following lysis, 50 *μ*g of each sample was separated by SDS-PAGE, and transferred to a nitrocellulose membrane then probed for RIPK1 and RIPK3 (1:1000) and signals detected by enhanced chemiluminescence according to the manufacturer's instructions. Relative protein levels were normalized to *β*-actin and, calculated using Image J and Microsoft Excel.

### Ultrastructure and immunogold electron microscopy

As previously described,^80^ after fixation in glutaraldehyde/paraformaldehyde (2.5%/1%), RCC and NK organ cultures were subjected to 1% osmium ferrocyanide for 1h, dehydrated in an ascending series of ethanol solutions, and embedded in Spurr's resin. Fifty nanometer sections were stained with uranyl acetate and lead citrate. For immunogold staining, as previously described, sections were stained for pMLKL^Ser358^ or pDrp1^Ser616^ and mitochondria (1:5) overnight and further incubated with 5 and 15 nm gold particles (British Biocell) (1:100), stained with uranyl acetate and lead citrate before viewing in a Hitachi Capital (UK) PLC, Leeds West Yorkshire at an accelerating voltage of 80 kV.

### Statistical analyses

All data represent mean±S.E.M. of *n*=3 independent experiments from at least six different patient organ cultures unless otherwise stated. Differences between two groups were analyzed by Student's *t*-test, and between >2 groups by one-way ANOVA followed by Bonferroni's *post hoc* *t*-test in GraphPad Prism version 5.02 (La Jolla, CA, USA). *P*<0.05 was regarded significant.

## Figures and Tables

**Table 1:** Expression of necrosomal signaling components regulated by wtTNF, R1TNF and R2TNF in malignant tubular epithelial cells in human RCC grade 1 organ cultures

	**UT**	**wtTNF**	**R1TNF**	**R2TNF**	**Subcellular localization**	**Pattern of immunostaining**
RIPK1	+/−	++++	++++	+/−	Cytoplasmic	Diffuse
RIPK3	+/−	++++	++++	+	Cytoplasmic/nuclear	Diffuse
Total MLKL	++++	++++	++++	+	Cytoplasmic	Diffuse
pMLKL^Ser358^	+/−	++++	++++	+	Cytoplasmic/nuclear	Diffuse/punctate
Total Drp1	++++	+++	+++	+	Cytoplasmic	Diffuse
pDrp1^Ser616^	−	+++	+++	+	Cytoplasmic	Punctate
pDrp1^Ser637^	++	+/−	+/−	+	Cytoplasmic	Punctate

Abbreviations: pMLKL^Ser358^, phosphorylated MLKL at Ser358; pDrp1^Ser616^, phosphorylated Drp1 at Ser616 and pDrp1^Ser637 -^phosphorylated at Ser637; RIPK1, receptor-interacting protein kinase 1; RIPK3, receptor-interacting protein kinase 3; Scores: − no labeling; +/− occasional positive labeling (>2%); + weak labeling (>5%); ++ intermediate labeling (>10%); +++ strong labeling (>40%); ++++ very strong labeling (>60%).

**Table 2:** Percentage of TNF-mediated cell death of tubular epithelial cells in human RCC grade 1 and adjacent non-tumor kidney (NK) organ cultures subjected to TUNEL

	**RCCoC**			**NKoC**		
**Treatment**	**wtTNF**	**R1TNF**	**R2TNF**	**wtTNF**	**R1TNF**	**R2TNF**
UT	4.60±0.13	5.1±0.10	4.5±0.3	2.7±0.3	2.2±01	2.1±0.16
alone	72.0±0.11^***^	70.2±0.12^***^	7.0±0.2*	61.0±0.6^***^	59.1±0.1^***^	5.0±0.12*
+n	39.1±0.31^+^	42.8±0.21^+^	5.2±0.3^┼^	41.1±0.3^+^	28.1±0.34^+^	3.2±0.15^┼^
+z	62.0±0.40^+^	60.7±0.07^+^	6.5±0.3	52.3±0.70^¥^	47.4±0.1^¥^	4.1±0.47
+n+z	28.1±0.17^**^	35.7±0.32^**^	5.0±0.2	36.1±0.17^#^	21.3±0.21^#^	3.0±0.23

Abbreviations: n, necrostatin-1 (10 *μ*M); NKoC, normal kidney organ culture; RCCoC, RCC organ culture; z, zVAD.fmk (20*μ*M); n+z (necrostatin-1 + zVAD.fmk); UT, Untreated. wtTNF (10 ng/ml), R1TNF and R2TNF (1 *μ*g/ml) were used. Percentage represents mean±S.E.M. (*n*=6); ^***^P<0.0001, *P<0.001 *versus* UT; ^+^*P*<0.001, ^+^*P*<0.01 *versus* wtTNF and R1TNF, ^┼^*P*<0.05 *versus* R2TNF; ^¥^*P*<0.05 *versus* wtTNF or R1TNF (NKoC); ^**^P<0.05 *versus* wtTNF+N (RCCoC); ^#^*P*<0.05 *versus* wtTNF or R1TNF (NKoC).

## Abbreviations

RCC: renal clear cell carcinoma
NK: normal human kidney
TNF: tumor necrosis factor
TNFR1: tumor necrosis factor receptor 1
TNFR2: tumor necrosis factor receptor 2
UT: untreated
wtTNF: wild-type tumor necrosis factor
R1TNF: TNFR1-selective mutein
R2TNF: TNFR2-selective mutein
RIPK1: receptor-interacting protein kinase 1
RIPK3: receptor-interacting protein kinase 3
MLKL: mixed lineage kinase domain
pMLKL^Ser358^: Mixed lineage kinase domain phosphorylated at Serine 358
Drp1: Dyamin-related protein 1
pDrp1^Ser637^: Dyamin-related protein 1 phosphorylated at Serine 637
pDrp1^Ser616^: Dyamin-related protein 1 phosphorylated at Serine 616
FADD: fas-associated protein with a death domain
TRADD: TNFR-associated protein with a death domain
c-Flip: cellular FLICE-inhibitory protein
mTECs: malignant tubular epithelial cells
MNCs: infiltrating mononuclear cells
VECs: vascular endothelial cells
Nec-1: necrostatin-1
NSA: necrosulfonamide
zVAD.fmk: carbobenzoxy-valyl-alanyl-aspartyl-[O-methyl]- fluoromethylketone
PDTC: pyrrolidine diothiocarbamate
NF*κ*B: nuclear factor *κ*B
PGAM5: phosphoglycerate mutase 5
PLA: proximity ligation assay
TUNEL: Terminal deoxynucleotidyl transferase dUTP nick end labeling
