# Supplementary material for: Tubular epithelial cells in renal clear cell carcinoma express high RIPK1/3 and show increased susceptibility to TNF receptor 1-induced necroptosis
Source: Cell Death Dis. 2016 Jun 30;7(6):e2287–. doi: 10.1038/cddis.2016.184 (PMC5108336; doi:10.1038/cddis.2016.184)
Supplement: Supplementary Information [file cddis2016184x1.doc]

**Supplementary Methods**

***In situ* Proximity Assay (PLA)**

We used PLA assay to investigate the effect of wtTNF, R1TNF and R2TNF on interaction of RIPK3 and RIPK1 or pMLKL^Ser358^ in organ cultures from human RCC grade 1 and NK. 5μm-thick de-wax sections were rinsed in phosphate buffered saline (PBS) containing 0.01% Tween-20 (PBST) and blocked for 1h at room temperature (RT) with blocking buffer [10% Fetal Calf Serum (FCS) in PBST; FCS/PBST]. This was followed by incubation at 4^o^C overnight with mouse anti-RIPK3 and rabbit anti-RIPK1 or rabbit anti-pMLKL^Ser358^ all used at 1:100 dilution. Sections were then washed in Duolink II Wash Buffer-A for 2 (x5 min); proximity ligation was performed using the Rabbit PLUS and Mouse MINUS Duolink *in situ* PLA kits, ligation and Amplification stocks (OLINK Bioscience) according to the manufacturer's instructions. Subsequently, slides were washed in Duolink II Wash Buffer-B, dehydrated, rinsed in MilliQ water and mounted in DAPI-containing Vectashield mounting medium (Vector Laboratories, Peterborough, UK). Negative control included omission of the primary antibodies or the ligase. Also, a control assay was performed on single stained parallel sections.

***In Situ* Hybridization (ISH)**

5μm-thick de-wax sections of human RCC grade 1 and NK organ cultures either untreated or TNF-treated were fixed in freshly prepared 4% formaldehyde in 0.1 mol/L PBS for 5 min at room temperature (RT). After washing in two changes of PBS, sections were treated with 5μg/ml Proteinase-K (Roche Diagnostics, East Sussex, UK) in PBS for 8 min at RT. Enzyme activity was inhibited by incubation with 2mg/ml glycine in PBS for 5 min and rinsed in 2X sodium citrate-buffered saline (SSC) for 5 min. Slides were then pre-hybridized at 37°C for 1h in hybridization buffer [1 mol/L Tris-HCI, pH 7.4, 20X SSC, 0.05 mol/L EDTA, pH 8.0, 50% dextran sulfate, 50X Denhart’s solution, 50% formamide and DEPC-H_2_O]. All reagents were of molecular biology grade from Sigma-Aldrich, Gillingham, UK. Excess solution was removed and 100μl of hybridization buffer containing digoxigenin-labeled anti-sense human RIPK1 (NM_003804) or human RIPK3 (NM_006871) (~4.6μg/ml) (MW Biotech-AG, UK) was added to each section and incubated overnight in a humidified chamber at 37°C. Post-hybridization washings were performed as follows: 2X SSC for 5 minutes at 37°C; 0.2X SSC, 60% formamide for 5 minutes at 37°C with two changes; 2X SSC for 5 min at RT with two changes; and in 100 mmol/L Tris-HCI, 150 mmol/L NaCI, pH 7.5 (TBS) for 5 min at room temperature. The sections were then processed for immunological detection. Briefly, non-specific binding was blocked by incubating sections in blocking buffer (10% fetal calf serum in PBS with 0.02% Tween-20; FCS/PBST) for 30 min at RT. Excess blocking solution was removed and 100μl of 1:200 dilution in 10% FCS/PBST of sheep anti-digoxigenin F(ab)_2_ antibody conjugated to alkaline phosphatase (Roche Diagnostics) was added to sections and incubated in a humidified chamber at RT for 2h. Following washings in PBST for 5 min with two changes, antibody-binding sites were visualized using 100μl of color substrate (alkaline phosphatase ready-to-use substrate kit containing β-chloroindolyl phosphate and nitroblue tetrazolium; NBT-BCIP) (Sigma-Aldrich). The sections were then washed thoroughly in Milli-Q water and mounted in Glycergel Aqueous Mounting Medium (Dakocytomation). Additional controls to confirm specificity of probe-binding included using digoxigenin-labeled Oligo-dT or actin cocktail probes from R&D systems, and negative controls included incubation of parallel sections with a sense probe to either RIPK1 or RIPK3 (MW Biotech). Images were captured using a Leitz Laborlux 1212 Research Microscope with Infinity 2 camera.

#### Combined TUNEL and IHC

5μm-thick de-wax sections of untreated and TNF-treated organ cultures from human RCC grade 1 and NK organ cultures were incubated with 50μg/mL Proteinase-K (Roche Diagnostics) pH 7.5 for 8 min in RT. Sections were washed in Milli-Q water and exposed to TdT buffer [containing 200mM potassium cacodylate, 25mM Tris-HCI, 0.25mg/m bovine serum albumin (BSA), 5mM cobalt chloride, pH 6.6] for 5 min, and incubated in a moist chamber with a mixture of TUNEL label [containing flourescein-11-dUTP] and 0.8U/µl TdT enzyme for 45 min at 37°C. For negative controls, TdT enzyme was omitted. Sections were then washed in TB buffer [containing 30mM sodium citrate, 300mM sodium chloride] for 15 min in RT, rinsed with Milli-Q water, and incubated in PBST for 10 min. This was followed by incubation of sections with anti-pMLKL^Ser358^ or pDrp1^Ser616^ or anti-pDrp1^Ser637^ (~1:100 dilution) overnight at 4^o^C followed by anti-rabbit-AlexaFluor^568^ and 1µg/ml Hoechst 33342 for nuclei detection. Sections were then mounted in Vectashield mounting medium and images captured using a Leica confocal laser scanning microscopy.

**Supplementary Figure Legends**

**Supplementary Figure 1. TUNEL on human RCC grade 1 and adjacent non-tumor kidney (NK).** (**A**) In contrast to untreated cultures (UT) which show a rare ^TUNEL+^mTEC, wtTNF- and R1-TNF-treated cultures induced an increased number of ^TUNEL+^mTECs (arrows). R2-TNF-treated cultures show comparable level of ^TUNEL+^mTECs as UT cultures. Blue nuclei counterstained with Hoechst 33342. (**B)**. Quantification of ^TUNEL+^TECs by paired Student’s *t*-test; data are presented as percentage of dead TECs. UT or R2-TNF vs R1-TNF or wtTNF: ^***^p<0.0001-,^*^p<0.05- vs UT; ^┼^p<0.001 vs wtTNF or R1TNF; ns-not significant. Bars= Mean ± SEM; Results are representative of n=3 independent 6 organ cultures experiments with similar results.

**Supplementary Figure 2. Hematoxylin and eosin stained sections of human RCC grade 1-4 (FG1-4) and adjacent non-tumor kidney (NK).** Sections of NK demonstrate remarkable morphology with intact, well-defined tubules and glomeruli In comparison, sections of RCC grade 1 (FG1) (***a***) show small round tumor cells with a clear cytoplasm surrounded by a distinct cell membrane and contain round and uniform nuclei with inconspicuous or absent nucleoli. RCC grade 2 (FG2) (***b***) shows tumor cells with medium-sized nuclei with slightly irregular contours and often with small but distinct nucleoli and punctate areas of necrosis within a tumor nodule. RCC grade 3 (FG3) (***c***) show irregular larger nuclei with prominent nucleoli (at x100 magnification) and large areas of necrosis within tumor zone. RCC grade 4 (FG4) (***d***) showed tumor cells with pleomorphic open chromatin or hyperchromatic scattered around extensive areas of necrosis, with nuclear material embedded within necrotic zones (arrow) (***e, f***). Tubular epithelial cells (*t*); Glomeruli (Glom); Magnifications: *a*-x100; *b-e*-x400; (*f) zoomed x2.3.*

**Supplementary Figure 3. Combined-immunofluorescence of RIPK1 or RIPK3 and cytokeratin in organ cultures of human** **RCC grade 1.** Untreated cultures (UT) show negligible levels of RIPK1 and RIPK3 expression (*green*) with the signal seen only in infiltrating mononuclear cells (MNCs; arrowheads) and in a few mTECs (arrows). In contrast, wtTNF- and R1TNF treatment induced an increased level of both proteins, co-localized with cytokeratin (CK) (*red*) in mTECs (shaded **arrows**), with RIPK3 detected in both cytoplasm and nuclear (open arrows). R2TNF-treatment also induced RIPK1 and RIPK3 expression but to a lesser extent than wtTNF and R1TNF (arrows). Results are representative of n=3 independent 6 organ cultures experiments with similar results. mTECs- malignant tubular epithelial cells; CK-Cytokeratin; nuclear counterstained with Hoechst 33342.

**Supplementary Figure 4** (**A**). **Combined-immunofluorescence for RIPK1 or RIPK3 and Cytokeratin in organ cultures of non-tumor kidney (NK).** In contrast to untreated cultures (UT) which show a rare signal for RIPK1 and RIPK3 wtTNF- and R1-TNF-treated cultures show a marked signal for both proteins in normal tubular epithelial cells (TECs -*green*), also positive for cytokeratin (CK-*red*) (**arrows**), with both RIPK1 and RIPK3 also detected in some infiltrating MNCs (open-arrows). R2-TNF-treated cultures show a rare signal of RIPK1 and RIPK3, comparable to UT cultures. (**B**). Mean Fluorescence intensity (MFI) for RIPK1 and RIPK3 in human RCC grade 1 and NK organ cultures. Nuclei stained with Hoechst 33342; original magnifications - x40 & x63; ^**^p<0.01-vs UT, ^*^p<0.05-vs UT, ±p<0.05 vs TNF or R1TNF, ns-not significant. Results are representative of n=3 independent 6 organ cultures experiments with similar results. TECs-tubular epithelial cells.

**Supplementary Figure 5.** **Representative photomicrographs of *in situ* hybridization for RIPK1 and RIPK3 in organ cultures of RCC grade 1 and** **adjacent non-tumor kidney (NK). (A)** In RCC cultures, wtTNF induce a strong mRNA for both kinases in malignant tubular epithelial cells (TECs-arrows) as compared to R2TNF, which induce a weak to moderate signal. (**B**) In NK cultures, wtTNF-induce a moderate signal for RIPK1 and RIPK3 in normal TECs (t; white arrows), in peritubular capillaries (black arrows) and in infiltrating MNCs within interstitium and glomeruli (Glom) (black arrowheads) while R2TNF induce mRNA for both kinases mainly on infiltrating MNCs (black arrowheads) and peritubular capillaries (black arrows), with a weak signal on a few normal TECs (t; white arrows). Results are representative of n=3 independent 6 organ cultures experiments with similar results. Magnifications; x400.

**Supplementary Figure 6. (A) Immunohistochemical staining for RIPK1 and RIPK3 on sections of RCC grade 1 and adjacent non-tumor kidney (NK) organ cultures treated with TNF.** Representative photomicrographs show a strong brown reaction product of 3’3-diaminobenzidine (DAB) substrate for RIPK1 and RIPK3 on sections incubated with the primary antibody in both the study groups (^+^TNF^-^peptide). Pre-incubation of the primary antibodies with a corresponding blocking peptide (^+^TNF^+^peptide) resulted in near complete block of the signal in NK, and complete block in RCC. Haematoxylin was used for nuclear staining (blue color). Glom-glomerular, t-tubules, Magnification-x200. (**B**). siRNA gene knockdown studies in the human cell line HEK293, after 48 h of transfection, the cells were treated with treated with zVAD-fmk (50μM) and Smac Mimetic (100μM) prior to TNF (10ng/mL) for 4 h to induce necroptosis show a marked expression of RIPK1 and RIPK3 in cells treated with control siRNA (non-targeting siRNAs, SMARTpool) and in comparison to a diminished level of expression in cells treated with siRNA to RIPK1 or RIPK3. Images are representative of n=3 independent experiments from 3 separate organ culture experiments with similar results.

**Supplementary Figure 7. Immunohistochemical staining for RIPK1 and RIPK3 on sections of RCC grade 1 and adjacent non-tumor kidney (NK) organ cultures treated with TNF (with or without PDTC; a specific NFκB inhibitor).** TNF-induced expression of RIPK1 and RIPK3 in both the study groups; ***panel A***- (NK cultures) shows intense signal of the kinases in mainly in glomerular and in normal TECs, not blocked by PDTC, ***panel B*** - (RCC cultures) shows strong signal mainly confined to mTECs (arrows), not blocked by PDTC. ***In panel B***, PDTC failed to block TNF-induced increase in the kinases. ***In panel C -*** control sections of RCC cultures show TNF-induction of NFκBp65^p-ser276^ expression and a diminished signal in PDTC-treated cultures. t-tubules, TEC-tubular epithelial cells, Glom-glomerular. Images are representative of n=3 independent experiments from 3 separate organ culture experiments with similar results. Magnifications-x200.

**Supplementary Figure 8. Immunohistochemical staining for active caspase 8^p18^, NFκBp65^p-ser276^** **and c-FLIP in RCC grade 1 and adjacent non-tumor kidney (NK) organ cultures treated with TNF** (**A**). Untreated cultures of NK show absence of active caspase 8^p18^ and NFκBp65^p-ser276^ but a strong signal for c-FLIP mainly in nTECs (t), and glomeruli (Glom). In contrast, TNF-induced a strong signal of active caspase 8^p18^ in resident mononuclear cells (arrows), NFκBp65^p-ser276^ and c-FLIP in nTECs (t). (**B**). In comparison to NK, untreated RCC cultures show presence of active caspase 8^p18^ in MNCs with a weak infrequent signal also seen in some mTECs (orange arrow) in TNF-treated cultures. Similarly, an infrequent signal for NFκBp65^p-ser276^ was evident in mTECs in untreated RCC, with a similar pattern and but with increased intensity in TNF-treated cultures (arrows). A strong signal for c-FLIP (arrows) was detected in untreated cultures with no obvious change in TNF-treated cultures. t-tubular epithelial cells, Glom-glomerular. representative of n=3 independent experiments from 3 separate organ culture experiments with similar results. Magnifications-x200.

**Supplementary Figure 9. Confocal images of total MLKL and total Drp1 in organ culture of** **RCC grade 1.** A strong signal of both proteins is seen mainly confined to malignant tubular epithelial cells (arrows), with no obvious differences between untreated cultures (UT) and cultures treated with wtTNF, R1TNF or R2TNF. Results are representative of n=3 independent experiments from 6 separate organ culture experiments with similar results. CK-pan-cytokeratin. Magnifications-x40.

**Supplementary Figure 10**. (**A**) **Immunohistochemical staining for pMLKL^Ser358^ in RCC grade 1 and adjacent non-tumor kidney (NK) organ cultures treated with TNF.** Representative photomicrographs show a strong brown reaction product of 3’3-diaminobenzidine (DAB) substrate for pMLKL^Ser358^ on sections incubated with the primary antibody in both the study groups (^+^TNF^-^peptide). Pre-incubation of the primary antibodies with corresponding blocking peptide (^+^TNF^+^peptide) resulted in a diminished signal. Haematoxylin was used for nuclear staining (blue color). Glom-glomerular, t-tubules. Images are representative of n=3 independent experiments from 3 separate organ culture experiments with similar results. Magnification-x200. (**B**). siRNA gene knockdown studies in the human cell line HEK293, after 48 h of transfection, the cells were treated with zVAD-fmk (50μM) and Smac Mimetic (100μM) prior to TNF (10ng/mL) for 4 h to induce necroptosis show a marked expression of RIPK1 and RIPK3 in cells treated with control siRNA (non-targeting siRNAs, SMARTpool) and in comparison a diminished level of expression in cells treated with siRNA to MLKL or Drp1. Magnification-x40.

**Supplementary Figure 11. Effect of wtTNF, R1TNF and R2TNF on pMLKL^Ser358^, pDrp1^Ser616^ and pDrp1^Ser637^ expression in organ cultures** **of** **RCC grade 1 and adjacent non-tumor kidney (NK).** (**A**) wtTNF-induced a marked expression of pMLKL^Ser358^ and pDrp1^Ser616^ but a reduced signal for pDrp1^Ser637^ compared to R2TNF-treated cultures, with signal mainly confined to malignant tubular epithelial cells (mTECs-arrows). Magnifications-x40. (**B**) Mean fluorescent intensity (MFI) for the phosphorylated proteins in normal TECs and mTECs, which show a statistical significant difference between the cultures. Bars=Mean ± SEM; Results are representative of n=3 independent experiments from 6 separate organ culture experiments with similar results;^***^p<0.001-UT, ^*^p<0.05- vs UT; ^┼^*p*<0.05- vs wtTNF or R1TNF (NKoC); ns- not significant.

**Supplementary Figure 12. *In Situ* Proximity Ligation Assay show wtTNF-induce an interaction between RIPK1-RIPK3 and RIPK3-pMLKL^Ser358^ in organ culture of RCC grade 1.** In comparison to R2TNF-treated cultures, wtTNF induced a strong interaction of RIPK1-RIPK3 and RIPK3-pMLKL^Ser358^ in malignant tubular epithelial cells (mTECs) appearing as strong red fluorescence spots (**A**). Each individual interacting protein pair observed as a red spot by confocal microscopy is expressed as the number of signals/cell (PLA spots/cell). Magnifications-x40. (**B**) Quantification of the PLA spots in TECs in the two study groups; RCC (RCCoC) and adjacent non-tumor kidney (NK) organ cultures (NKoC) show a statistically significant difference between the study groups, more pronounced in RCCoC. Bars=Mean ± SEM. Results are representative of n=3 independent experiments from 6 separate organ culture experiments with similar results;^***^p<0.001- vs UT, ^**^p<0.01- vs UT, ^*^p<0.05- vs UT, ±p<0.05- vs NKoC; ns-not significant.

**Supplementary Figure 13. Representative confocal images of pMLKL^Ser358^ or pDrp1^Ser637^ and TUNEL in organ cultures of** **RCC grade 1 and NK.** Compared to R2TNF-treated cultures, wtTNF induced increased levels of ^TUNEL+^mTECs (*green*) associated with (**A**) pMLKL^Ser358^ (*red*). Magnification-x40. (**B, C**). Quantification of ^TUNEL+^mTECs/pMLKL^Ser358+^ and ^TUNEL+^mTECs/pDrp1^Ser616+^ show statistically significant differences between the cultures. Bars=Mean + SEM and representative of n=3 independent experiments from 6 separate organ culture experiments with similar results. ^***^p<0.0001-; ^**^p<0.01-; ^*^p<0.01 vs UT; ±p<0.05 vs wtTNF or R1TNF (NKoC); ns-not significant; TEC-tubular epithelial cells (m)-malignant.
