# Supplementary figures and images for: Tubular epithelial cells in renal clear cell carcinoma express high RIPK1/3 and show increased susceptibility to TNF receptor 1-induced necroptosis
Source: Cell Death Dis. 2016 Jun 30;7(6):e2287–. doi: 10.1038/cddis.2016.184 (PMC5108336; doi:10.1038/cddis.2016.184)

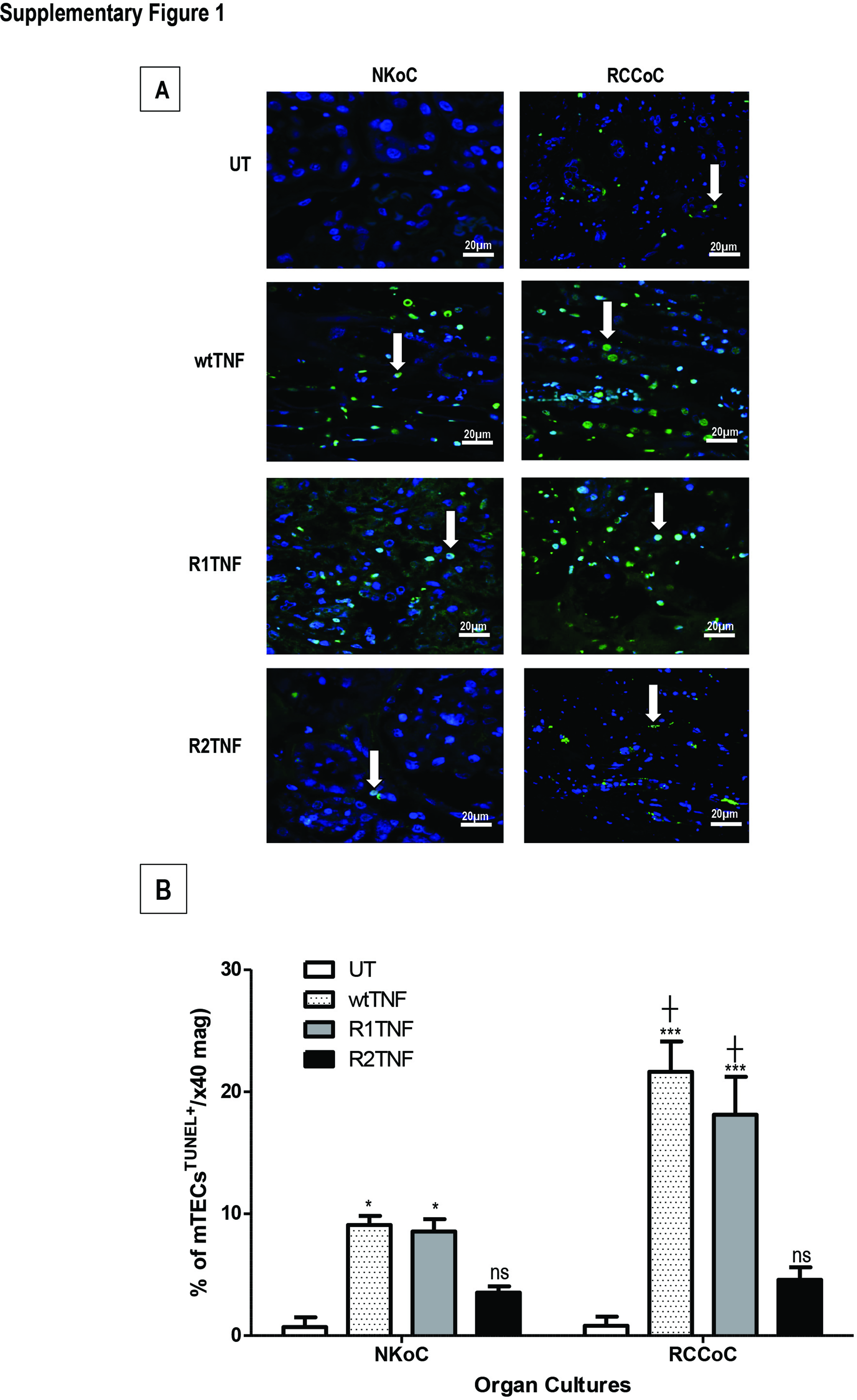

Supplement: Supplementary Figure 1 [file cddis2016184x2.tif]

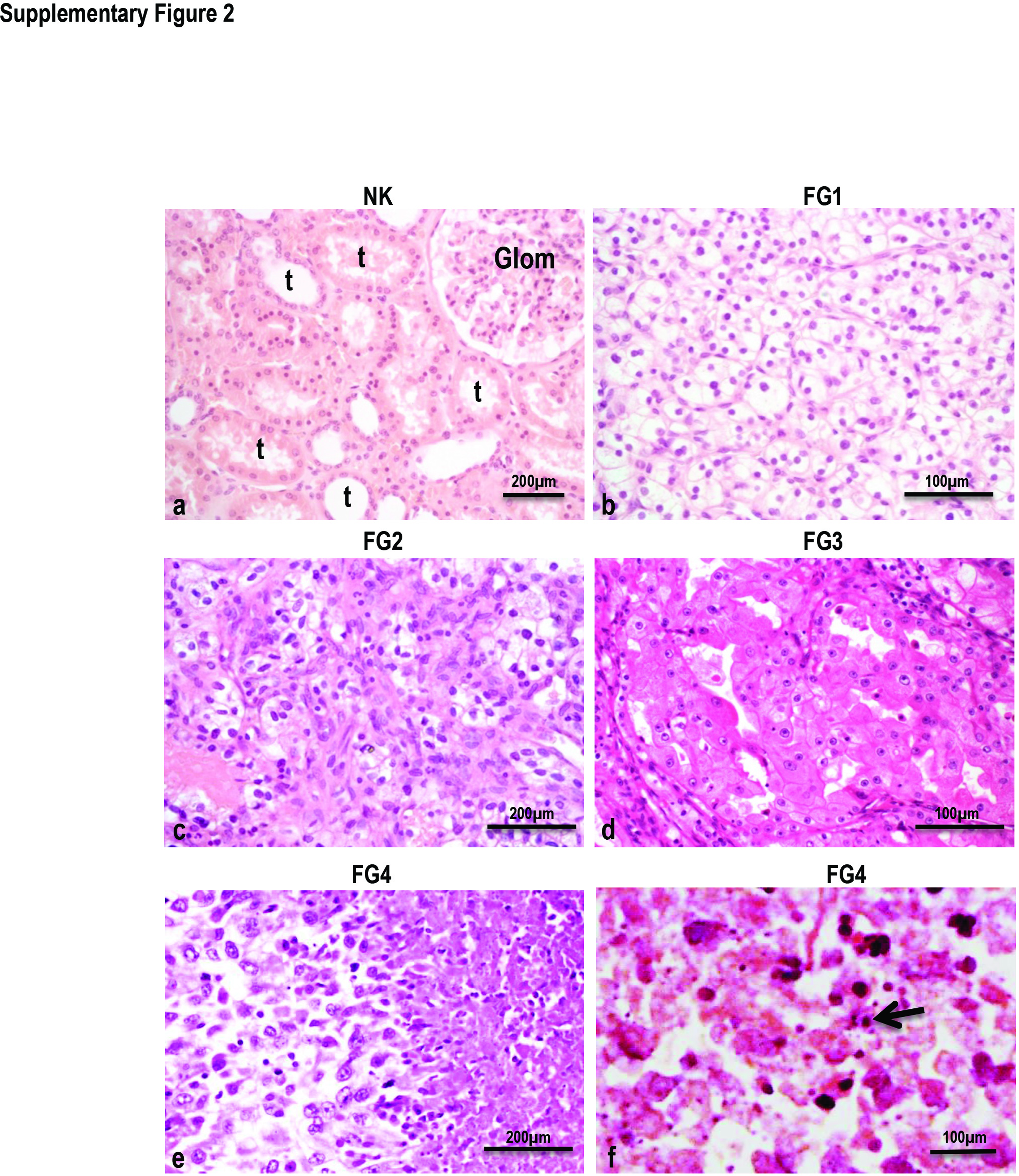

Supplement: Supplementary Figure 2 [file cddis2016184x3.tif]

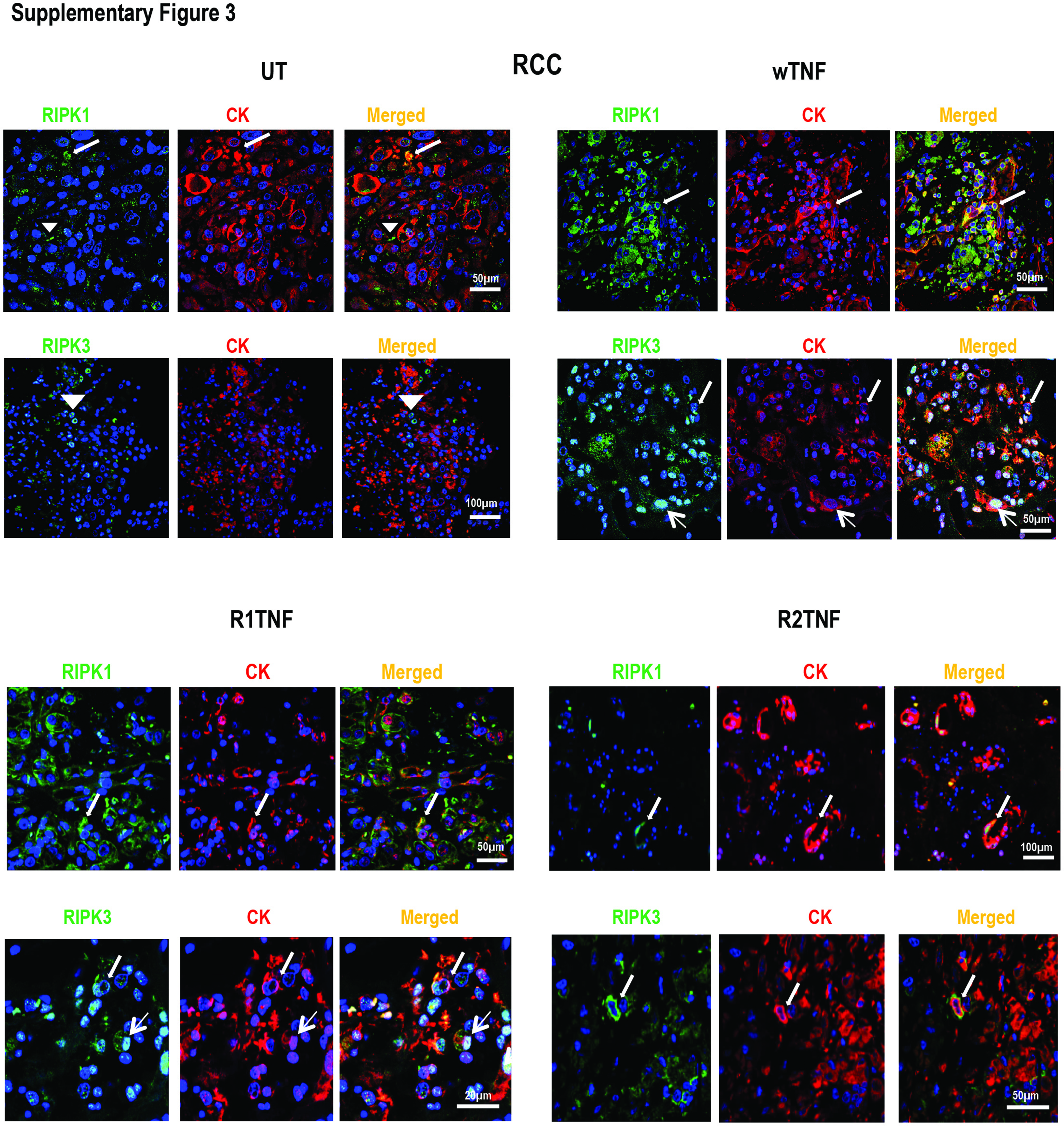

Supplement: Supplementary Figure 3 [file cddis2016184x4.tif]

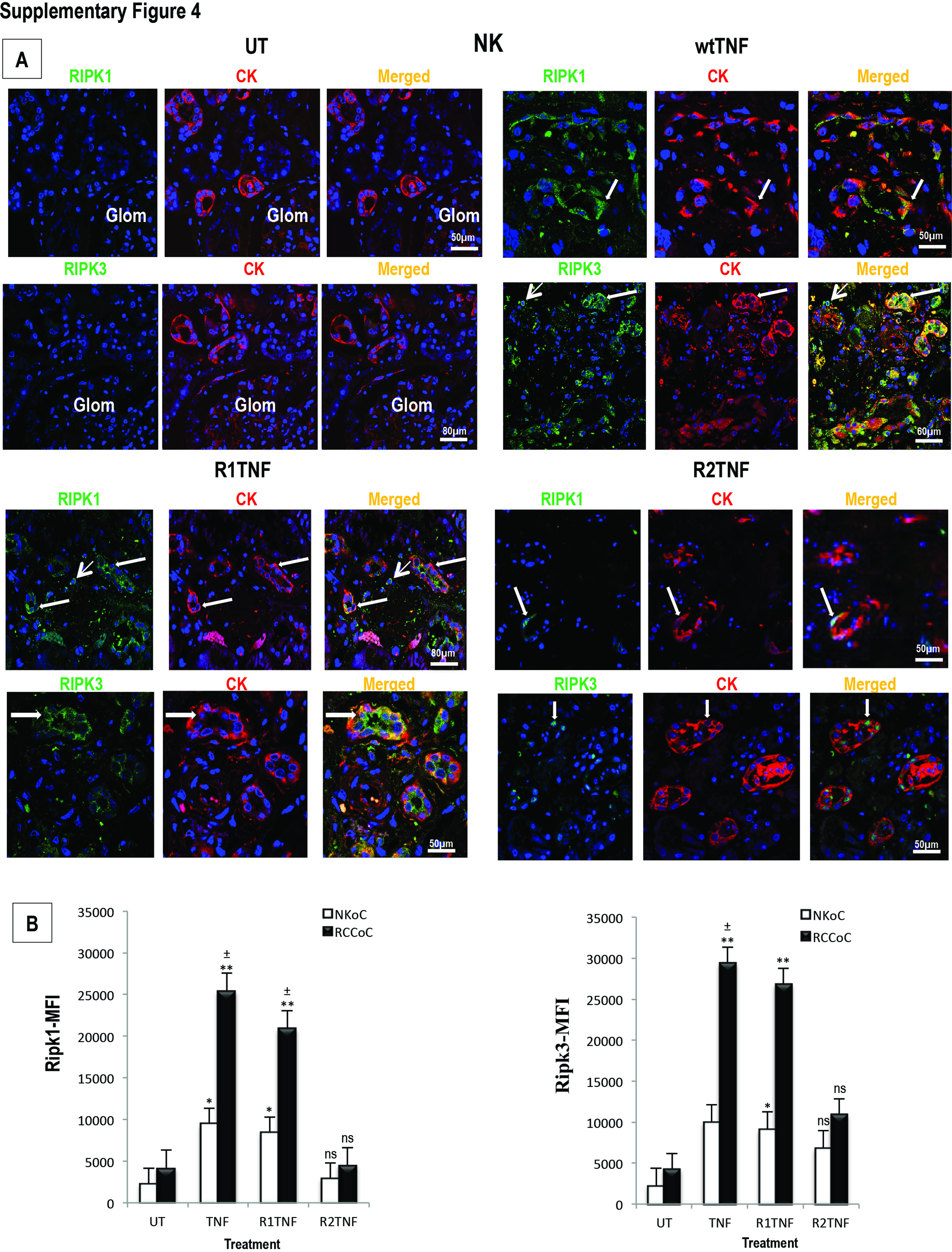

Supplement: Supplementary Figure 4 [file cddis2016184x5.tif]

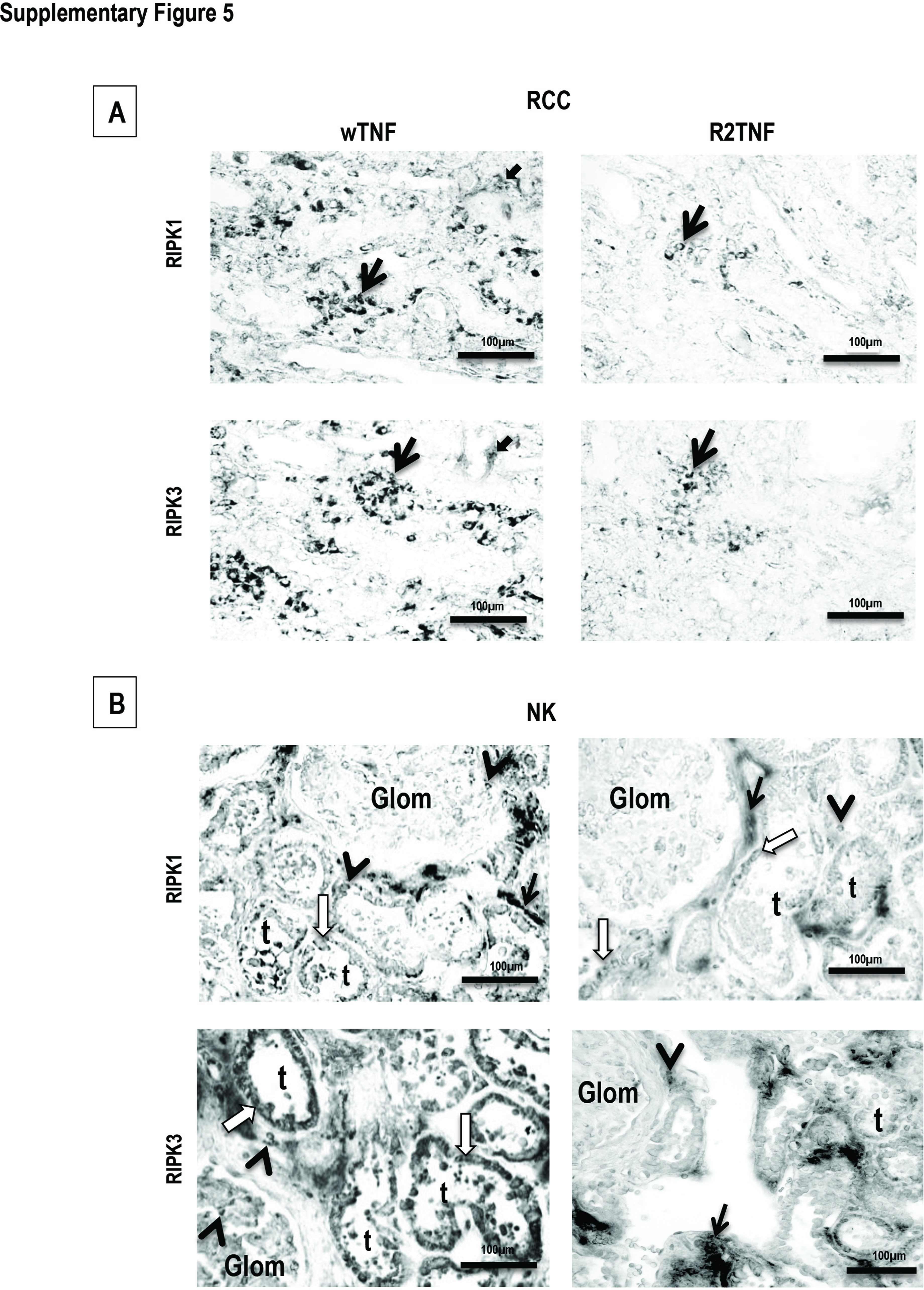

Supplement: Supplementary Figure 5 [file cddis2016184x6.tif]

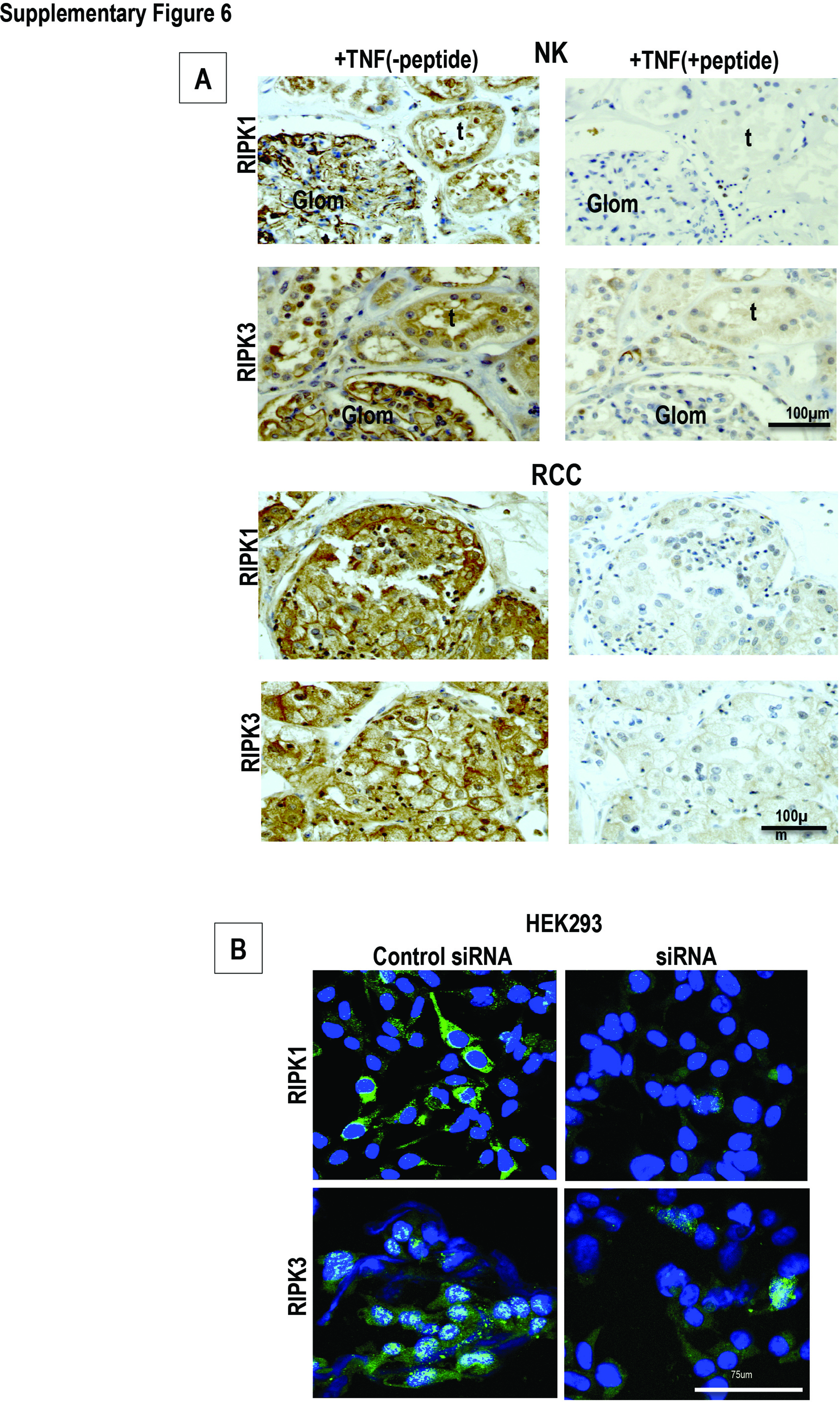

Supplement: Supplementary Figure 6 [file cddis2016184x7.tif]

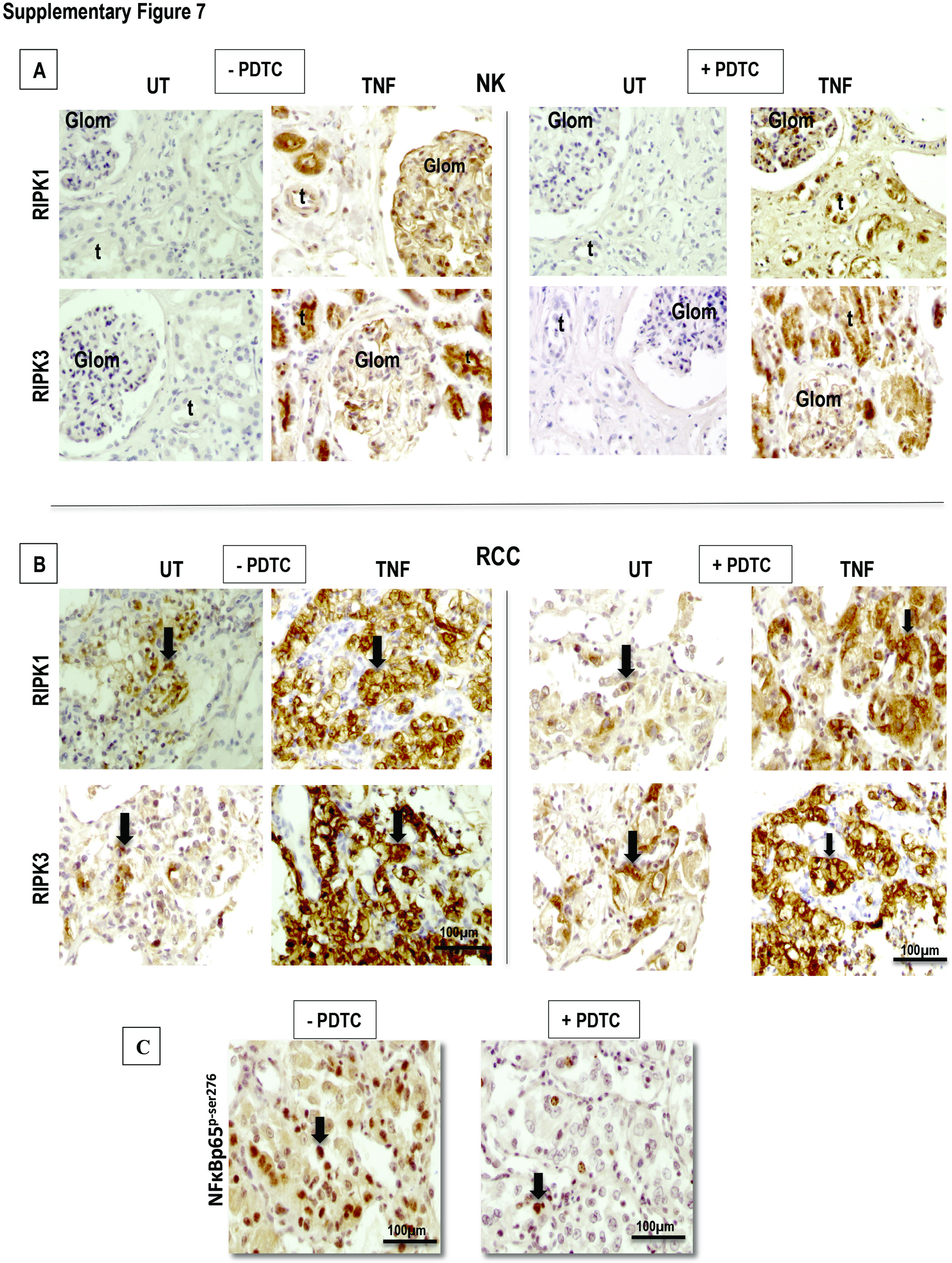

Supplement: Supplementary Figure 7 [file cddis2016184x8.tif]

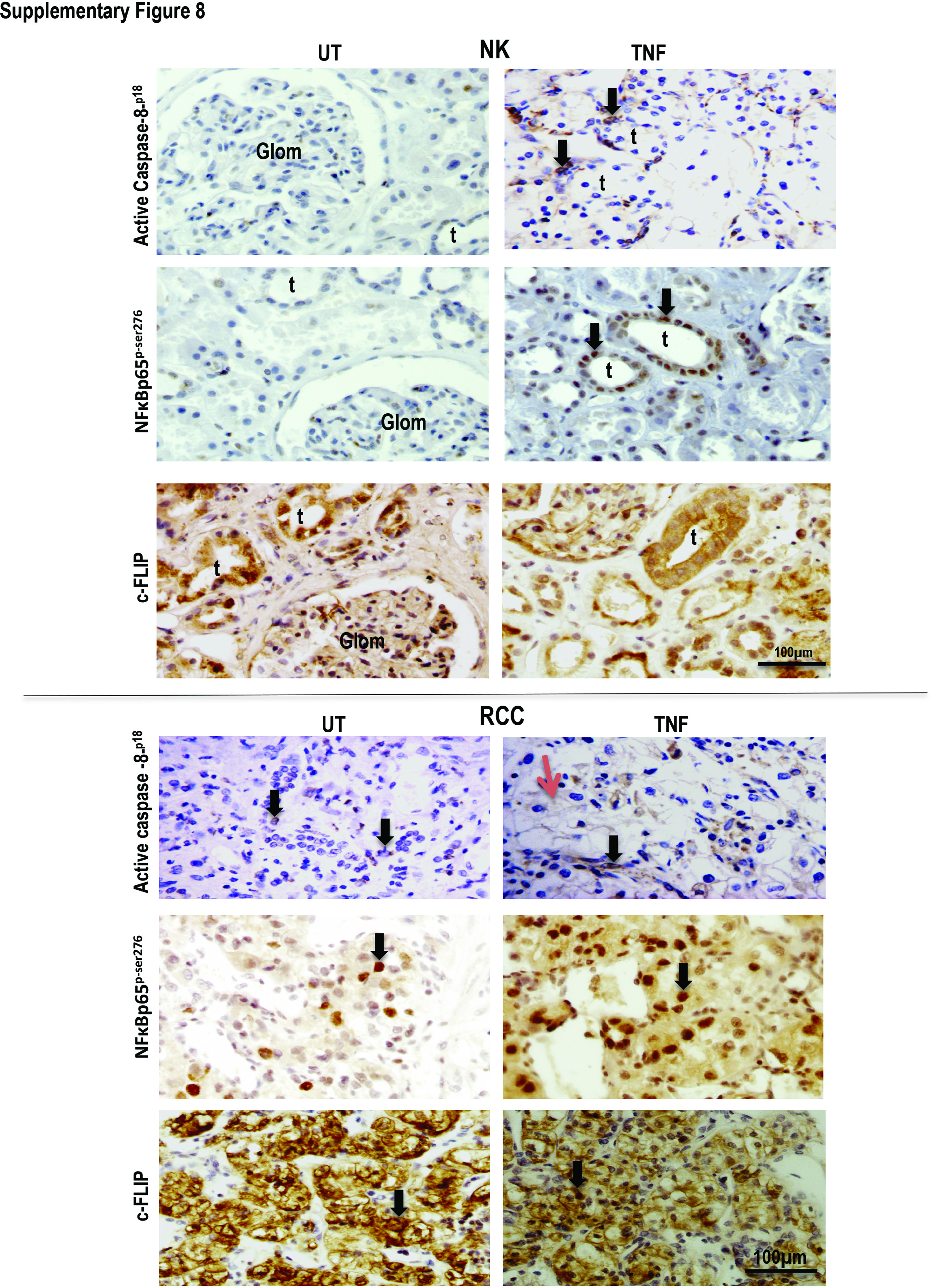

Supplement: Supplementary Figure 8 [file cddis2016184x9.tif]

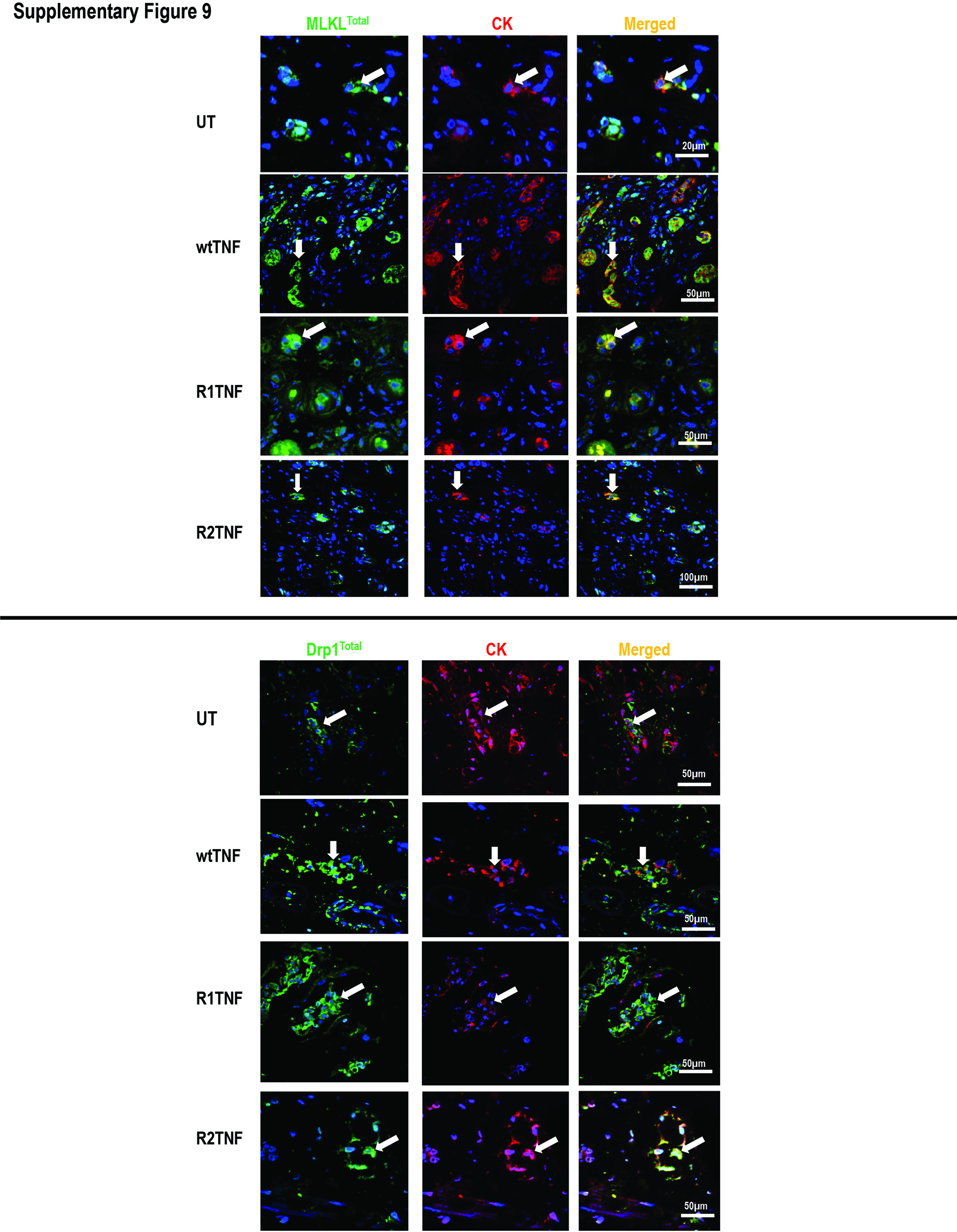

Supplement: Supplementary Figure 9 [file cddis2016184x10.tif]

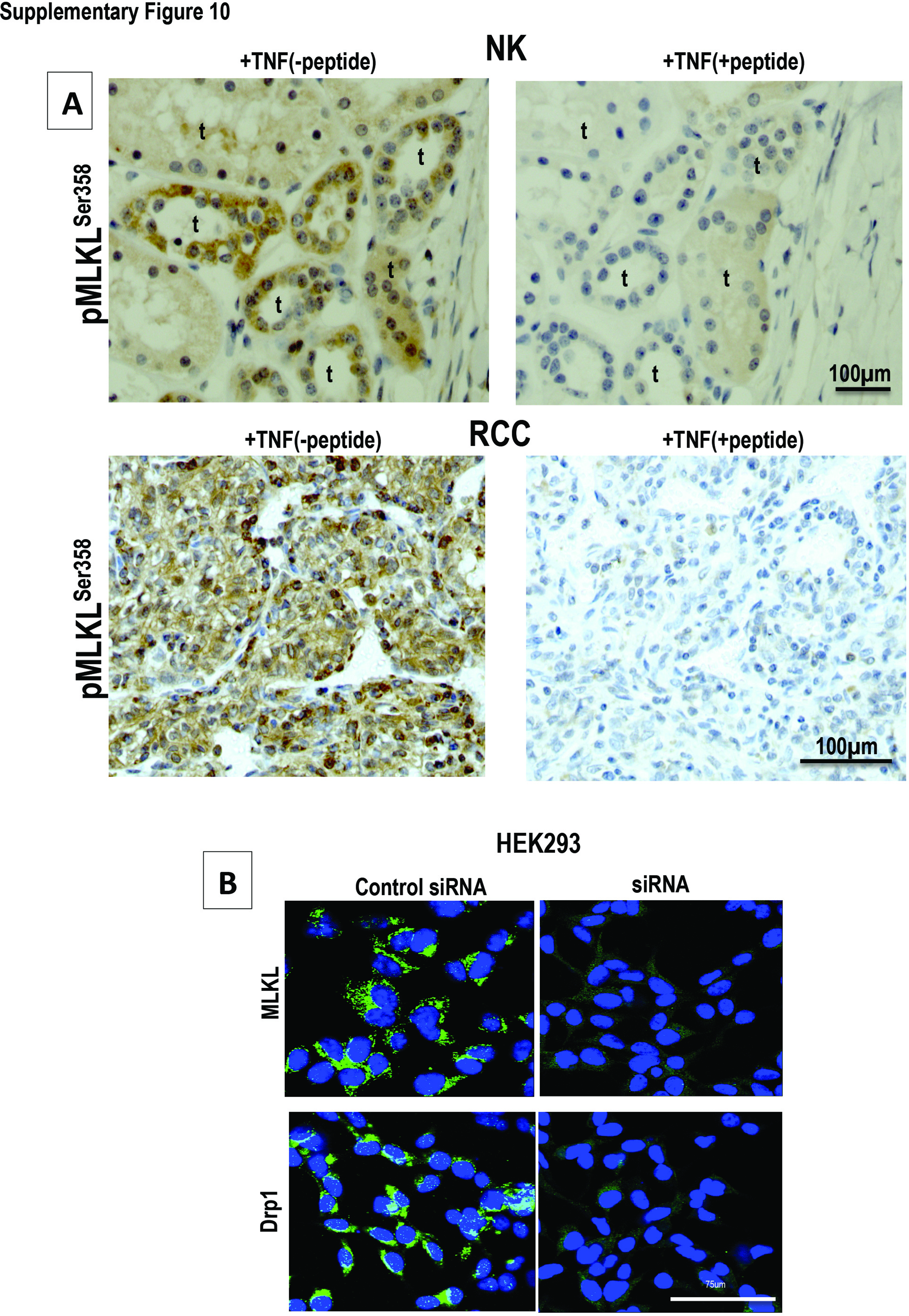

Supplement: Supplementary Figure 10 [file cddis2016184x11.tif]

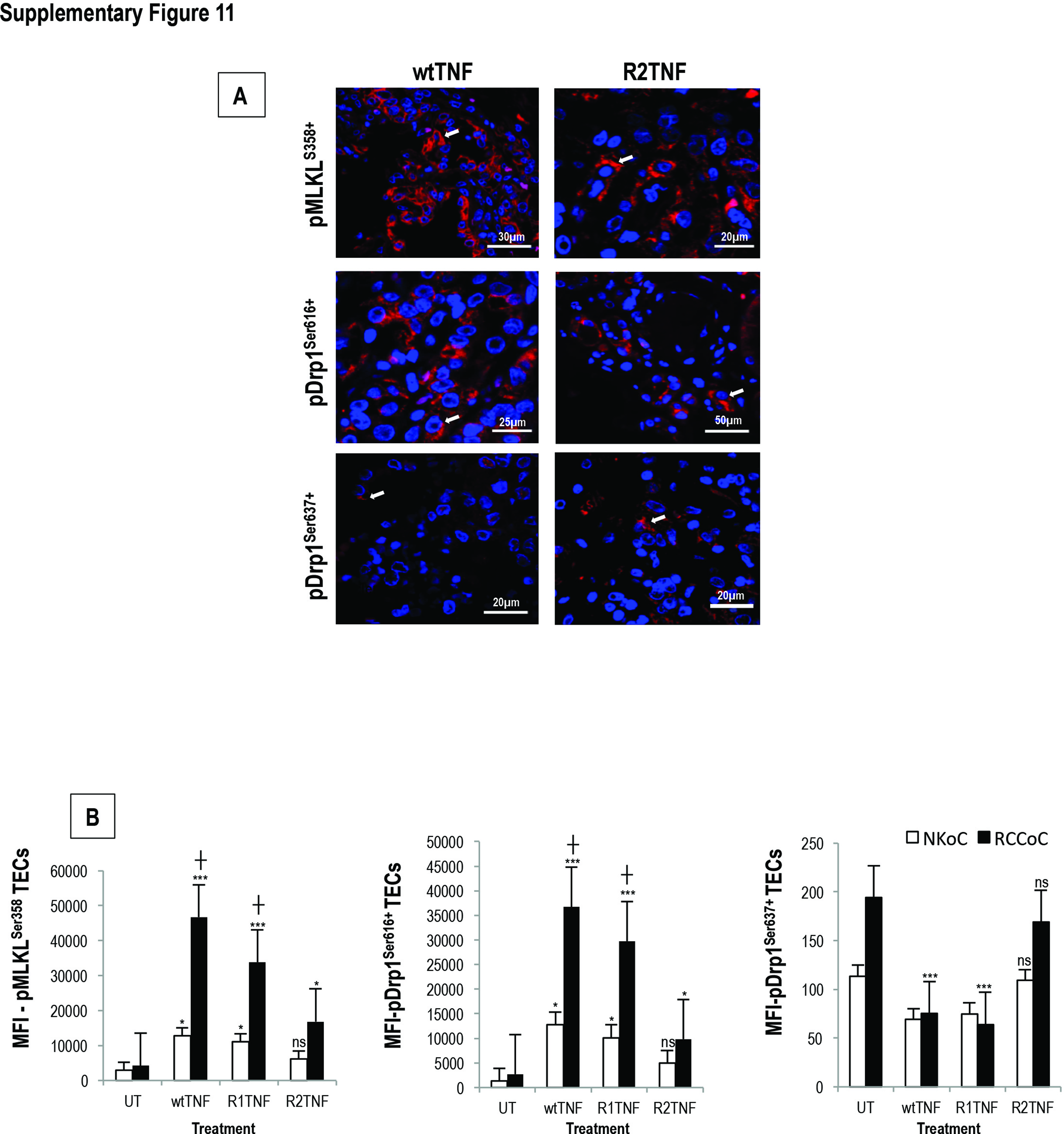

Supplement: Supplementary Figure 11 [file cddis2016184x12.tif]

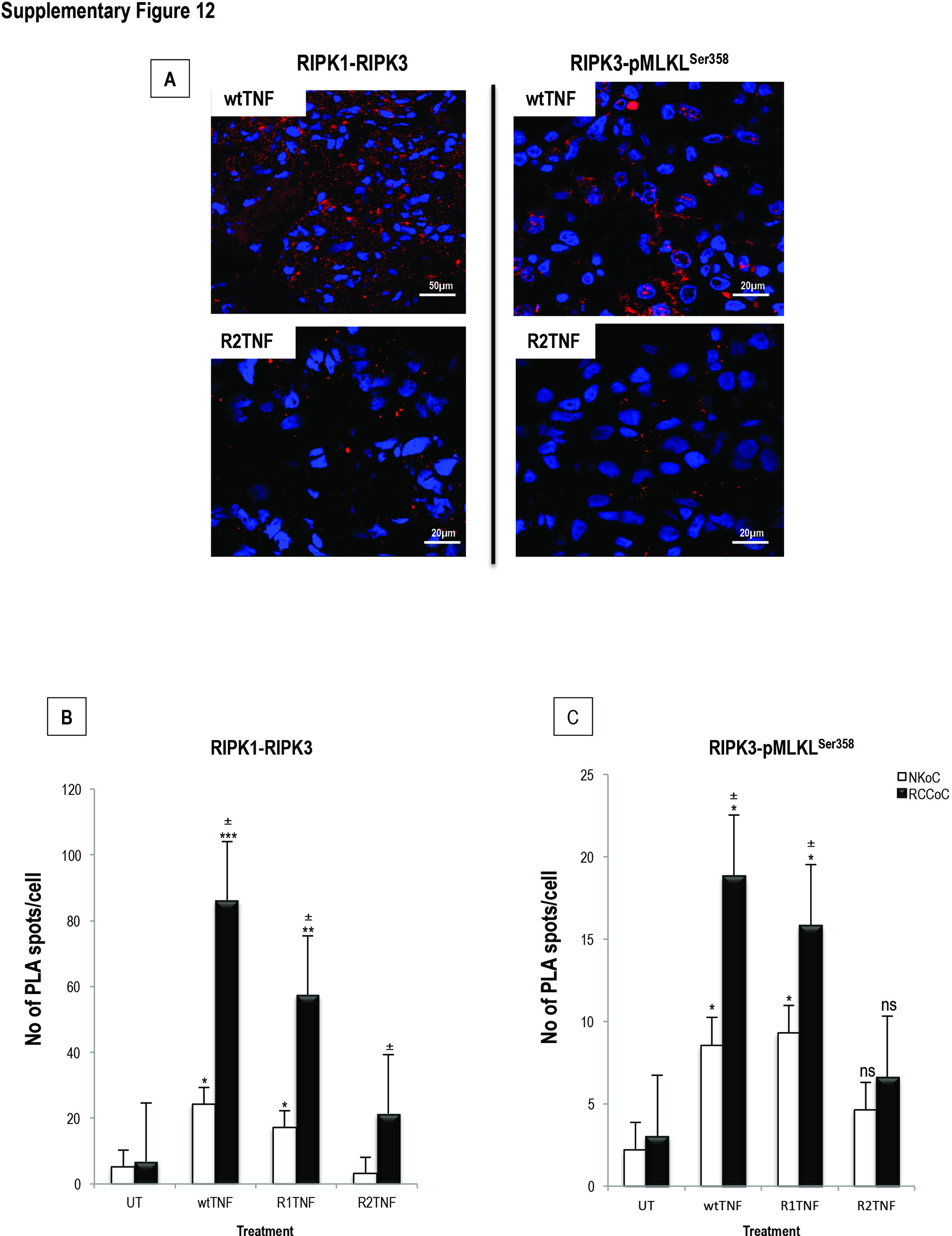

Supplement: Supplementary Figure 12 [file cddis2016184x13.tif]

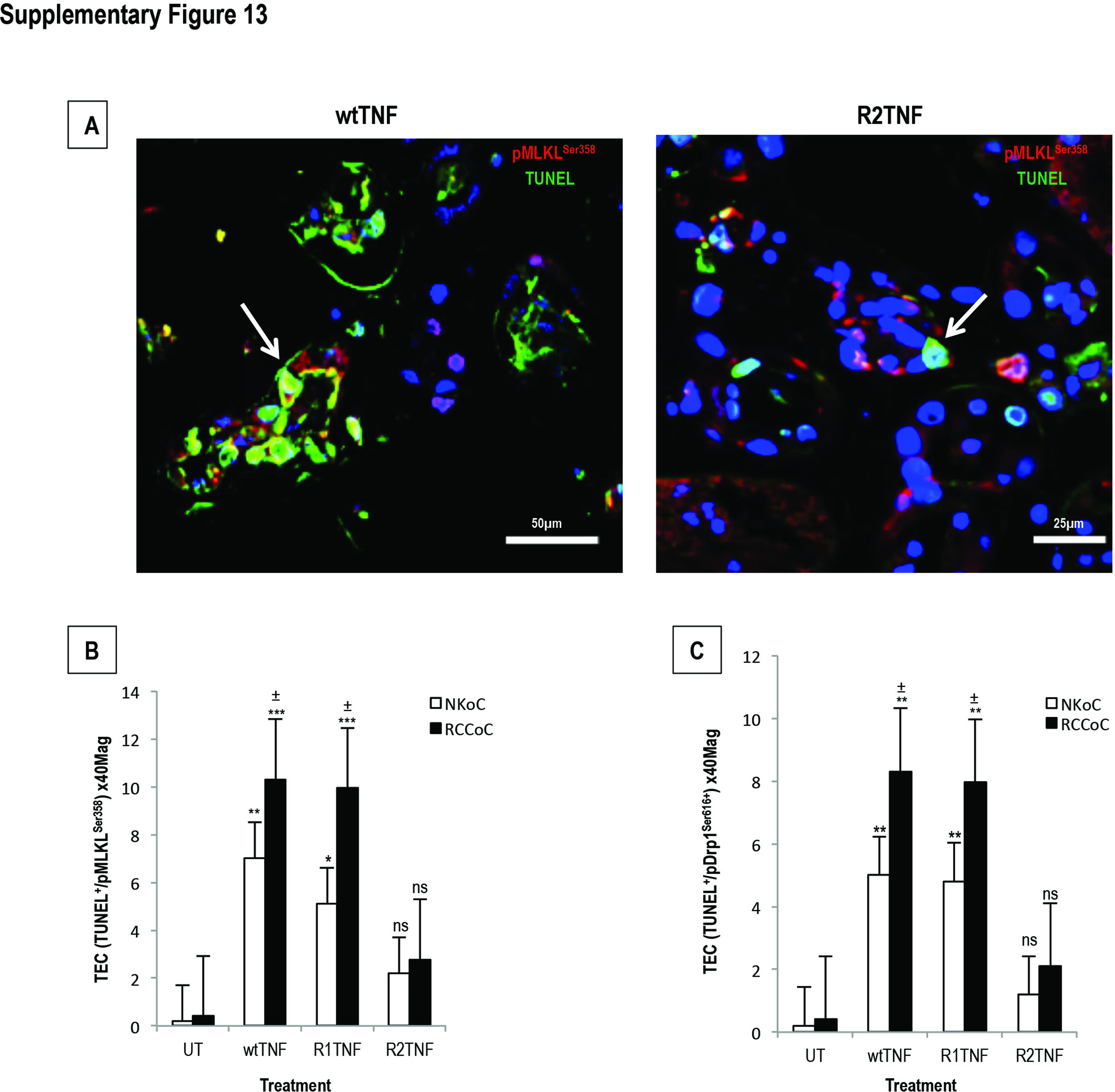

Supplement: Supplementary Figure 13 [file cddis2016184x14.tif]
